# Inference of Quantitative Models of Bacterial Promoters from Time-Series Reporter Gene Data

**DOI:** 10.1371/journal.pcbi.1004028

**Published:** 2015-01-15

**Authors:** Diana Stefan, Corinne Pinel, Stéphane Pinhal, Eugenio Cinquemani, Johannes Geiselmann, Hidde de Jong

**Affiliations:** 1 INRIA Grenoble – Rhône-Alpes, Grenoble, France; 2 Laboratoire Interdisciplinaire de Physique (LIPhy, CNRS UMR 5588), Université Joseph Fourier, Grenoble, France; Hellas, GREECE

## Abstract

The inference of regulatory interactions and quantitative models of gene regulation from time-series transcriptomics data has been extensively studied and applied to a range of problems in drug discovery, cancer research, and biotechnology. The application of existing methods is commonly based on implicit assumptions on the biological processes under study. First, the measurements of mRNA abundance obtained in transcriptomics experiments are taken to be representative of protein concentrations. Second, the observed changes in gene expression are assumed to be solely due to transcription factors and other specific regulators, while changes in the activity of the gene expression machinery and other global physiological effects are neglected. While convenient in practice, these assumptions are often not valid and bias the reverse engineering process. Here we systematically investigate, using a combination of models and experiments, the importance of this bias and possible corrections. We measure in real time and in vivo the activity of genes involved in the FliA-FlgM module of the E. coli motility network. From these data, we estimate protein concentrations and global physiological effects by means of kinetic models of gene expression. Our results indicate that correcting for the bias of commonly-made assumptions improves the quality of the models inferred from the data. Moreover, we show by simulation that these improvements are expected to be even stronger for systems in which protein concentrations have longer half-lives and the activity of the gene expression machinery varies more strongly across conditions than in the FliA-FlgM module. The approach proposed in this study is broadly applicable when using time-series transcriptome data to learn about the structure and dynamics of regulatory networks. In the case of the FliA-FlgM module, our results demonstrate the importance of global physiological effects and the active regulation of FliA and FlgM half-lives for the dynamics of FliA-dependent promoters.

## Author Summary

A wide variety of methods for the reverse engineering of regulatory networks and the identification of quantitative regulation functions are available. We investigate some common assumptions that are made in the application of these methods to time-series transcriptomics data, in the context of a central module in the motility network of *E. coli*. We show that these assumptions, which hypothesize that mRNA concentrations are good proxies for protein concentrations and that the gene expression machinery is equally active across different physiological conditions, are often not valid and may lead to biased inference results. We also show how models of gene expression can be used in combination with suitable experimental controls to correct for this bias and improve the inference process. The contribution of our work is thus not the addition of another method to the rich store of available reverse engineering algorithms, but lies in the critical examination of the information provided by the experimental data and new ways to exploit this information in the algorithms. The proposed approach is relevant for a wide range of applications using time-series transcriptomics data. For the motility system under study, it has underlined the importance of global physiological effects, the active degradation of the transcription factor FliA as well as the secretion of the anti-sigma factor FlgM for the network dynamics.

This is a *PLOS Computational Biology* Methods paper.

## Introduction

DNA microarrays, RNA sequencing, and other high-throughput technologies yield huge amounts of data on the state of the transcriptional program in bacterial cells in different growth conditions and genetic backgrounds, at different time-points in an experiment. The information on the (relative) RNA abundances thus obtained, representative of the activity of the genes, have fueled the development of methods for inferring regulatory interactions among genes. In essence, these methods try to explain the observed variation in the activity of one gene in terms of the variation in the activity of other genes. A large number of inference methods have been proposed in the literature and have been successful in a variety of applications, although a number of difficult problems remain (see [1–7] for reviews).

A major problem with the use of transcriptome data for the inference of regulatory interactions is that often the active regulator is not mRNA but protein. Although protein and mRNA concentrations are moderately correlated at steady state [8, 9], this is generally not the case when the two are considered dynamically over time. Due to the fact that proteins and mRNAs have different half-lives, their concentrations evolve on different time-scales. For instance, mRNA half-lives are typically on the order of a few minutes in bacteria [10], whereas most proteins are quite stable [11, 12]. The effect of rapid responses in gene expression, within a single generation, may thus give rise to proteins persisting over several generations, endowing the cell with a memory of past events [9, 13]. As a consequence, inference of regulatory interactions from time-series transcriptome data alone may potentially lead to spurious results. Although quantitative proteomics techniques have much advanced recently [14, 15], it is not yet possible to directly measure protein concentrations *in vivo* and in real time.

A second problem derives from the fact that the dynamics of gene expression are not only controlled by transcription factors, small regulatory RNAs, and other specific regulators, but also by global physiological effects influencing the rates of transcription and translation of all genes [16–19]. Large-scale differences in gene expression over time or across conditions may therefore not just derive from transcriptional regulatory interactions, but also reflect global changes in cellular physiology, notably the concentrations of (free) RNA polymerase and ribosome, gene copy number, and the size of amino acid and nucleotide pools. Ignoring such changes in the activity of the gene expression machinery, for example in experiments with important variations of the growth rate, may lead to the inference of spurious regulatory interactions [20, 21]. Unfortunately, concentrations of (active) RNA polymerase and ribosome, as well as many other global physiological parameters, are difficult to quantify in a direct way.

These problems for reverse engineering come from two basic, usually implicit assumptions on the biological processes under study: (i) mRNA abundance is a good proxy for protein concentrations and (ii) the gene expression machinery is equally active across different physiological conditions. Although the fact that these assumptions are often not valid has been broadly recognized, very little has been done to study the resulting bias in a systematic way. The aim of this paper is to propose a combined experimental and computational approach to show how these assumptions affect the inference of quantitative models of bacterial promoters from time-series gene expression data and to propose theoretically sound and practically useful procedures to correct for this bias and improve the inference process.

We will notably focus on the case of gene expression measurements obtained by means of fluorescent reporters [22]. These technologies, which have become widespread in recent years, allow the activity of genes to be monitored *in vivo* and in real time [23, 24]. Exploiting these data makes it possible to quantify the difference between mRNA and protein concentrations as well as global physiological effects. In short, if the half-lives of the proteins are available, the models used for deriving the activities of genes from fluorescence data can be integrated to yield estimates of protein concentrations [25]. The global physiological state of the cell can be estimated from the activity of a constitutively expressed gene [17, 18], that is, a gene whose expression is not controlled by any particular transcription factor, but only depends on the activity of the transcriptional and translational machinery [26]. To which extent does the integration of the above information into the inference procedure improve the identification results, both structurally and quantitatively?

In order to answer this question, we applied our methodology to a central module in the regulatory network controlling the synthesis of flagella and the chemotaxis sensing system in *Escherichia coli* [27–29]. This module comprises the FliA and FlgM transcription factors and their targets. FliA or *σ* ^28^ is a sigma factor which directs RNA polymerase to operons coding for the flagellar filament and the chemotaxis sensing system controlling the flagellar motor. The effect of FliA is counteracted by the anti-sigma factor FlgM. As a typical example of a FliA-dependent gene we study *tar*. This gene encodes the aspartate chemoreceptor protein Tar, which responds to a decrease of the aspartate concentration in the medium. Tar stimulates the phosphorylation of downstream response regulators binding the flagellar motor component [30, 31]. The FliA-FlgM module forms a check-point in the temporally-organized expression cascade. It is particularly well-suited as a gold standard for our purpose, since the interactions in this network have been well-studied and protein stability has been found to play an important role in its functioning.

We experimentally excited the FliA-FlgM module in a variety of wild-type and mutant conditions, in different growth media, and measured the transcriptional response of the genes. These data were used to systematically test the information required for the reliable inference of the regulatory interactions and quantitatively predictive models of gene regulation. In a first step, we found that the use of *fliA* and *flgM* promoter activities, instead of their protein concentrations, did not allow the regulatory interactions to be recovered. Moreover, a quantitative model identified from the data fails to account for the observed dynamics of the *tar* promoter in most conditions considered here. The introduction, in a second step, of global regulatory effects, measured by means of a reporter gene driven by a constitutive promoter, results in the expected structure of regulatory interactions. The fit of the quantitative model to the data, however, is only marginally improved. We therefore estimated in a third step the concentrations of FliA and FlgM from the observed promoter activities and physiologically plausible half-lives of the proteins. The model quantitatively reproduces the observed activity of *tar* across the different conditions much better and the estimated parameter values agree with the expected regulatory role of FliA. Additional simulation studies, in which we systematically varied the half-lives of the proteins and the importance of global physiological effects, show that these factors may be even more important in other regulatory networks, notably when involving transcription factors with half-lives longer than the (exceptionally short) half-lives of FliA and FlgM.

We conclude that for the reliable reconstruction of transcriptional regulatory networks in microorganisms it is important to monitor not only specific transcription factors, but also global effects imposed by the cellular physiology, and to take into account both short-term transcriptional responses as well as their long-term effects on protein abundance. We have presented and validated a practical approach to integrate information on protein concentrations and global regulatory effects into the network identification process. Since the proposed strategy does not depend on any specific network inference method, and can in principle be combined with data obtained from experimental techniques other than fluorescent reporter genes, the approach is applicable to a large variety of network inference problems in both prokaryotic and eukaryotic systems.

## Results

### Monitoring the transcriptional response of the FliA-FlgM module

**Figure 1:**
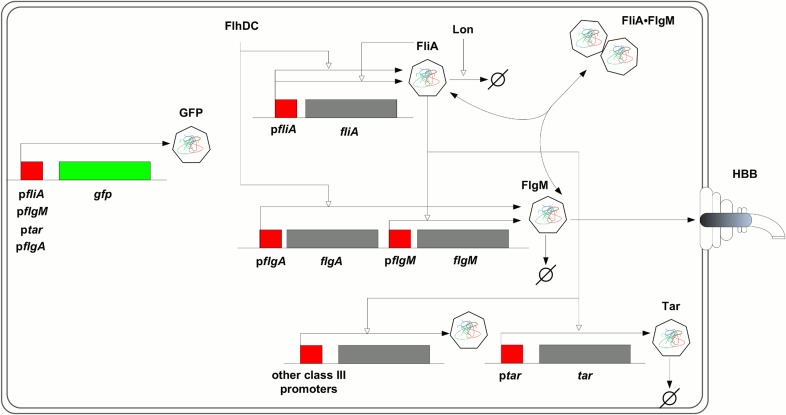
FliA-FlgM module. The regulatory circuit composed of the flagellar-specific transcription factor FliA, a sigma factor also known as *σ* ^28^, and the anti-sigma factor FlgM forms a check-point in the transcriptional hierarchy of the motility genes in *E. coli*. While *fliA* is transcribed from a single class 2 promoter (p*fliA*), *flgM* is transcribed from both a class 2 and a class 3 promoter (p*flgA* and p*flgM*, respectively). FliA binds to RNA polymerase core enzyme and directs transcription from a total of five class 3 promoters [33], including p*tar* and p*flgM*. When bound to FlgM, FliA cannot activate transcription. When the hook basal-body (HBB) structure is in place, however, FlgM is exported from the cell, thus releasing FliA from the inactive complex. FliA is subject to proteolysis by Lon, but FlgM-binding protects FliA from degradation. The *fliA* promoter is auto-regulated by FliA and by a number of other regulators, most importantly the motility master regulator FlhDC. The expression of FlhDC itself is under the control of a variety of regulatory factors, including RpoS, CpxR, and CsgD. The activity of the genes in the figure is measured by fusion of their promoters to a *gfp* reporter gene on a low-copy plasmid. Genes are shown in grey or green and their promoter regions in red. Regulatory interactions are represented by open arrows, association and dissociation of FliA and FlgM as well as degradation and export by filled arrows. The figure does not explicitly show that *fliA*, *flgM*, and *tar* are included in larger transcriptional units, the *fliAZY*, *flgAMN*, *flgMN* and *tar-tap-cheRBYZ* operons [33].

The more than 60 genes responsible for motility in bacteria are structured in a transcriptional hierarchy of three operon classes that has been mapped in detail for *Escherichia coli* and *Salmonella enterica* [27–29, 32]. The single class 1 operon *flhDC* encodes the proteins FlhD and FlhC, which form a heteromultimeric complex activating *σ* ^70^-dependent transcription of the class 2 operons. The latter encode the proteins making up the flagellar motor structure as well as a major regulator of the class 3 operons, the sigma factor FliA (*σ* ^28^). When bound to core RNA polymerase, FliA directs the transcription of the class 3 operons [33] that code for the proteins forming the filament structure of the flagellum and the chemotaxis sensing system. The aspartate chemoreceptor Tar is an example of such a class 3 protein. The action of FliA is counteracted by the anti-sigma factor FlgM, which binds to FliA and thus prevents its association with RNA polymerase. FlgM is encoded by the gene *flgM*, which is transcribed from both a class 2 promoter and a class 3 promoter. FlgM can be excreted from the cell through the center of the basal-body structure (Fig. 1).

The transcriptional hierarchy underlies a temporally-arranged order of events during the assembly of the flagella and the chemotactic sensing system [27–29, 32]. On the highest level of the hierarchy, the transcription of the flagellar master regulator responds to a variety of signals [34, 35]. For instance, the expression of the *flhDC* operon is repressed when the bacteria are grown on minimal medium with glucose [36]. When glucose is depleted from the environment, however, the signalling molecule cyclic AMP (cAMP) accumulates in the cell, inducing *flhDC* transcription through the intermediary of the cAMP receptor protein Crp [37]. In the presence of FlhDC, the class 2 operons, and thus the genes encoding the hook basal-body (HBB) structure as well as FliA and FlgM, are actively transcribed. FlgM sequesters FliA, and prevents it from transcribing the class 3 operons [38]. When the HBB structures have been completed, however, FlgM is secreted from the cell, releasing FliA and relieving the repression of the class 3 operons [38]. The FliA-FlgM interactions thus form a check-point in flagella formation, ensuring that the filament proteins are produced only when the basal body and the hook, to which the flagellar filaments are attached, are in place.

In order to investigate the regulation of the genes involved in this check-point, we measured the time-varying transcription of *fliA*, *flgM*, and *tar* (as an example of a class 3 gene) in *E. coli*. This was accomplished by means of fluorescent reporter systems, consisting of transcriptional fusions of a *gfp* reporter gene to the promoters of the target genes, carried on a low-copy plasmid. The strains transformed with the reporter plasmids were grown in 96-well microplates, following a previously-established protocol [17, 39, 40]. After an overnight preculture, the bacteria were diluted into fresh medium in the microplate and the absorbance of the cultures and the emitted fluorescence were monitored at 37°C in a thermostated microplate reader for 7 to 16 h, until growth arrest occurred. These kinetic experiments were carried out in different growth media (minimal M9 medium with glucose, rich LB medium) and in different genetic backgrounds (wild-type and deletion mutants of the global transcription regulators RpoS, CsgD, and CpxR) [40]. The timing and the strength of the induction of the hierarchy of motility genes varies among conditions, leading to a different time-varying excitation of the FliA-FlgM module.

While *fliA* and *tar* have a single promoter, this is not the case for *flgM*, which is transcribed from both a class 2 and a class 3 promoter, as discussed above. The fluorescence signal from the class 2 promoter, however, was found to be almost indistinguishable from background levels in all conditions (Text S7), consistent with the observation that most FlgM in the cell derives from the FliA-dependent promoter [29, 41]. In the analysis that follows, we therefore neglected *flgM* transcription from the class 2 promoter.

**Figure 2:**
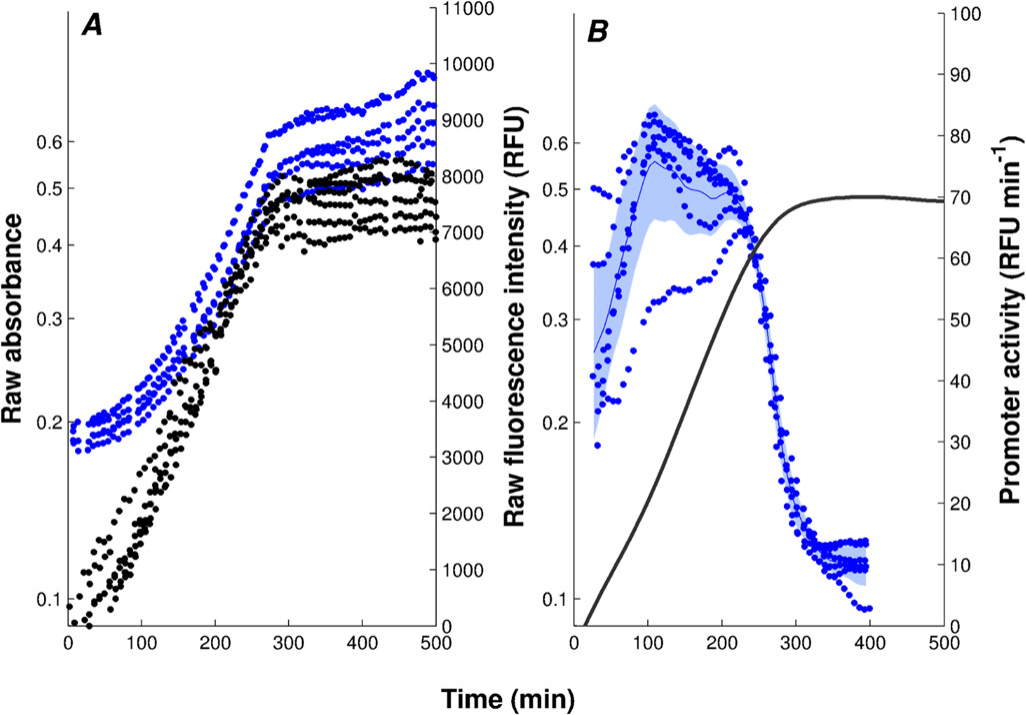
Primary data and promoter activities. *A:* Absorbance (•, black) and fluorescence (•, blue) data, corrected for background intensities, obtained with the Δ*cpxR* strain transformed with the p*tar*-*gfp* reporter plasmid and grown in M9 with glucose. *B:* Activity of the *tar* promoter, computed from the primary data as described in the *Methods and materials* and in Text S3. The solid black line corresponds to the mean of 6 replicate absorbance measurements and the shaded blue region to the mean of the promoter activities ± twice the standard error of the mean.

As illustrated in Fig. 2, and explained in more detail in the *Methods and materials* and Text S3, the primary absorbance and fluorescence signals can be transformed into promoter activities using kinetic models of gene expression. More precisely, the reporter gene data can be used to deduce protein synthesis rates [23, 25]. Under certain conditions, the latter are proportional to mRNA concentrations and promoter activities, and thus reflect the transcriptional activity of the gene (Text S2). Following established terminology, we will refer to the measured protein synthesis rates as promoter activities, or more generally, activities of genes.

**Figure 3:**
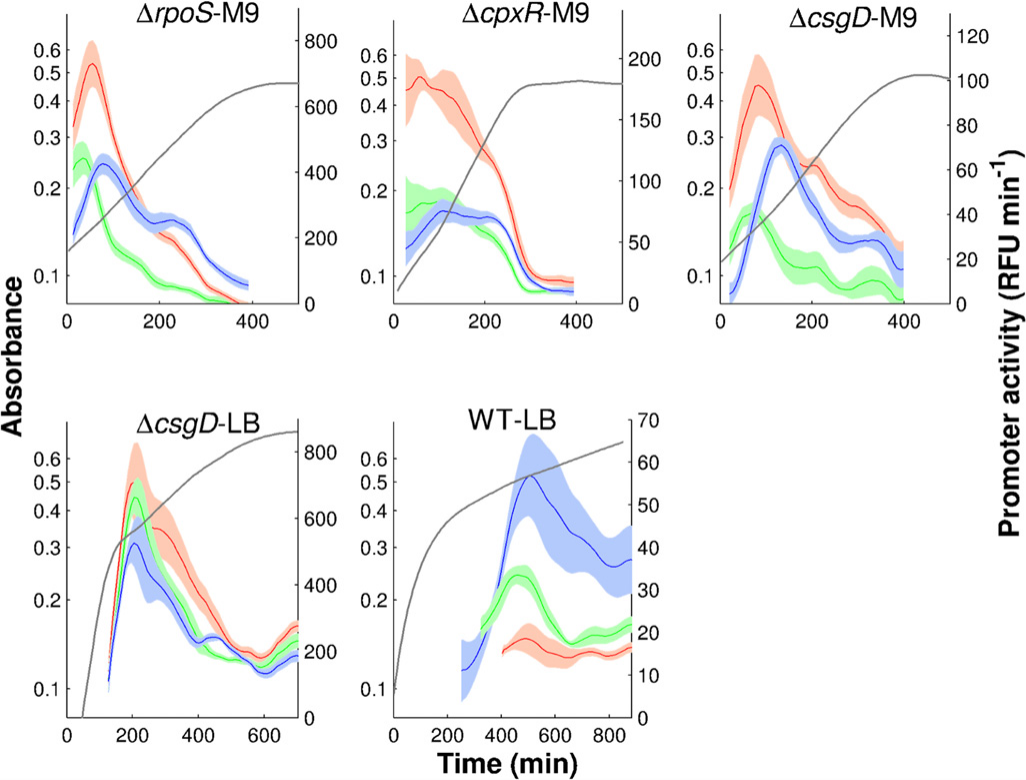
Promoter activities of genes in the FliA-FlgM module. The promoter activities of *fliA* (green), *flgM* (red), and *tar* (blue) measured by means of fluorescent reporter genes in the following experimental conditions: Δ*rpoS* strain grown in M9 (Δ*rpoS*-M9), Δ*cpxR* strain grown in M9 (Δ*cpxR*-M9), Δ*csgD* strain grown in M9 (Δ*csgD*-M9), Δ*csgD* strain grown in LB (Δ*csgD*-LB), and wild-type strain grown in LB (WT-LB). Grey lines report mean absorbance measurements in the various conditions. The promoter activities and absorbance profiles have been derived from the primary data as illustrated in Fig. 2.

In each of the experimental conditions, we have acquired 5 to 8 replicate measurements, which makes it possible to estimate the uncertainty in the derived promoter activities. Fig. 3 shows the results for the five conditions considered here: (i) Δ*rpoS* strain grown in M9 (Δ*rpoS*-M9), (ii) Δ*cpxR* strain grown in M9 (Δ*cpxR*-M9), (iii) Δ*csgD* strain grown in M9 (Δ*csgD*-M9),(iv) Δ*csgD* strain grown in LB (Δ*csgD*-LB), and (v) wild-type strain grown in LB (WT-LB). As expected [36], the fluorescence signals in the wild-type strain grown in minimal M9 medium with glucose were mostly indistinguishable from the background fluorescence and therefore this condition was not further considered. In one condition (WT-LB), the activities measured by means of reporter genes were validated using RT-qPCR (Text S6).

The measured promoter activities in Fig. 3 show some common features, such as a transient activity peak of the genes during exponential growth, followed by stabilization at a low level after growth arrest. The induction of the individual promoters has a distinct temporal order, corresponding to the level of the promoters in the transcriptional hierarchy [39]: *fliA*, *flgM*, *tar*. There are also clearly visible differences between the profiles across the conditions though. In M9 medium with glucose the motility genes in the mutant strains are transcribed right from the start, whereas in LB induction occurs only after a number of generations, consistent with previous reports [27, 36]. Moreover, the strength of induction and the duration of the activity peak varies from one condition to the other. For instance, the maximal activity of *tar* varies 10-fold between the WT-LB and Δ*csgD*-LB conditions.

### Identification of gene regulation functions from promoter activities

The circuit in Fig. 1 has been well-studied over several decades and its regulatory structure is well-known [27–29, 32, 33]. This makes it an excellent test case for investigating which information is required for the reliable inference of regulatory interactions and quantitative regulation models from time-series expression data. In a first step, we tested if we could account for measured time-varying promoter activities while ignoring the distinction between mRNA and protein concentrations as well as the activity of the gene expression machinery and other global physiological effects. This corresponds to the usual assumptions made in the analysis of transcriptome data.

**Figure 4:**
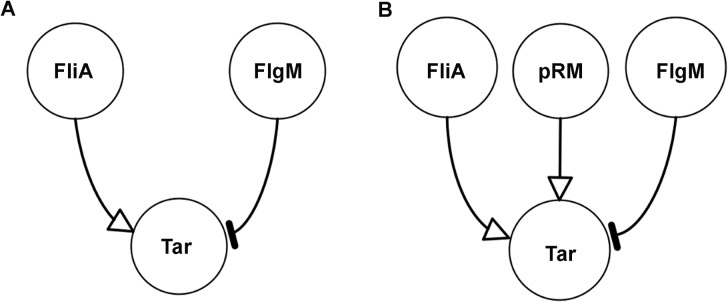
Pattern of regulatory interactions for *tar* and other class 3 genes. *A:* FliA activates and FlgM inhibits *tar*. *B:* Idem, but with global physiological effects, measured by the activity of the pRM promoter.

We expect FliA to be an activator and FlgM an inhibitor of target genes like *tar* and other class 3 genes (Fig. 4*A*). In order to check if this regulatory pattern is consistent with the reporter gene data, we used minimal sign pattern analysis [42]. This approach exploits time-series data to invalidate patterns of regulatory interactions, based on the assumption that the activity of a gene is a monotonic function of its regulators. For the patterns that remain after the invalidation step, so-called minimal sign patterns are computed, equivalent to the regulatory structures in Fig. 4. These patterns are minimal in the sense that removing any of the regulators results in an inconsistency with the data, while adding other regulators preserves consistency (see *Methods and materials* and Text S5 for details on the method).

We applied minimal sign pattern analysis to the reporter gene data in Fig. 3. In particular, we tested if the expected regulatory pattern in Fig. 4*A* is conserved when replacing the concentrations of FliA and FlgM by the measured promoter activities. We found that the activator role of FliA and the inhibitor role of FlgM are not consistent with the data. This is due to the fact that, over some interval of time in the condition Δ*rpoS*, a decrease of the promoter activity of *fliA* and an increase of the promoter activity of *flgM* coincide with an increase of the activity of *tar*. As a consequence, the sign pattern corresponding to the expected structure in Fig. 4*A* is rejected in the analysis.

Despite this structural problem, we also tested to which extent it is possible to quantitatively predict the activity of *tar* from the activities of their regulators. To this end, we developed a mechanistic model of the regulation of this promoter by FliA and FlgM. The model takes into account the titration of FliA by FlgM and the activation of transcription by (free) FliA. We made a quasi-equilibrium assumption for FliA-FlgM association and dissociation, justified by the fast time-scale on which these reactions occur in comparison with transcription and translation processes [43, 44]. Moreover, we chose a Hill function to describe promoter activation and included a basal synthesis rate. The resulting model is:

$$f(t)\;=k_{0}+k_{1}\frac{p_{A,free}{\left(t\right)}^{n}}{\theta^{n}+p_{A,free}{\left(t\right)}^{n}},$$ (1)

$$p_{A,free}(t)\;=\frac{1}{2}\left(-(K+p_{M}(t)-p_{A}(t))+\sqrt{{\left(K+p_{M}(t)-p_{A}(t)\right)}^{2}+4Kp_{A}(t)}\right),$$ (2)

where *f*(*t*) is the time-varying promoter activity, *p*_*A*,*free*_(*t*) is the concentration of free FliA, *θ* is a threshold constant for promoter activation, *k*_0_ and *k*_0_+*k*_1_ are the basal and maximal synthesis rates, respectively, and *n* is a Hill constant. The concentration of free FliA is computed from the concentrations *p*_*A*_(*t*) and *p*_*M*_(*t*) of total FliA and FlgM, respectively, and the FliA-FlgM dissociation constant *K*. All variables and parameters are non-negative and *n* ≥ 1. The concentration variables, as well as *θ* and *K*, have (arbitrary) units RFU, while the promoter activity and the rate constants have units RFU min^−1^. The derivation of the model is described in detail in Text S4. Notice that the model is in agreement with the expected pattern of regulatory interactions (Fig. 4*A*).

How well does this model fit the data when the total concentrations of FliA and FlgM in Eq. 2, *p*_*A*_ and *p*_*M*_, are replaced by the measured activities of *fliA* and *flgM*, respectively? We estimated the values of the kinetic parameters *c* = (*k*_0_,*k*_1_,*n*,*θ*,*K*) in the regulation model from the data obtained in all five conditions, using a multistart global optimization algorithm [45] to minimize the fitting error *Q*(*c*) (*Methods and materials*). The algorithm minimizes the mean-square error between the observed promoter activities and the predictions of the model of Eqs. 1–2, while taking into account differences in absolute promoter activity across conditions as well as the time-varying size of confidence intervals (*Methods and materials*). The parameters are chosen within physiologically plausible intervals. We notably require that the threshold *θ* lies within the range of observed FliA concentrations, which corresponds to making the assumption that within the conditions considered here, *tar* varies between its minimal and maximal activity. This is consistent with the observation that motility is low during exponential growth in LB medium [27, 36] and high in a Δ*csgD* strain [40].

**Figure 5:**
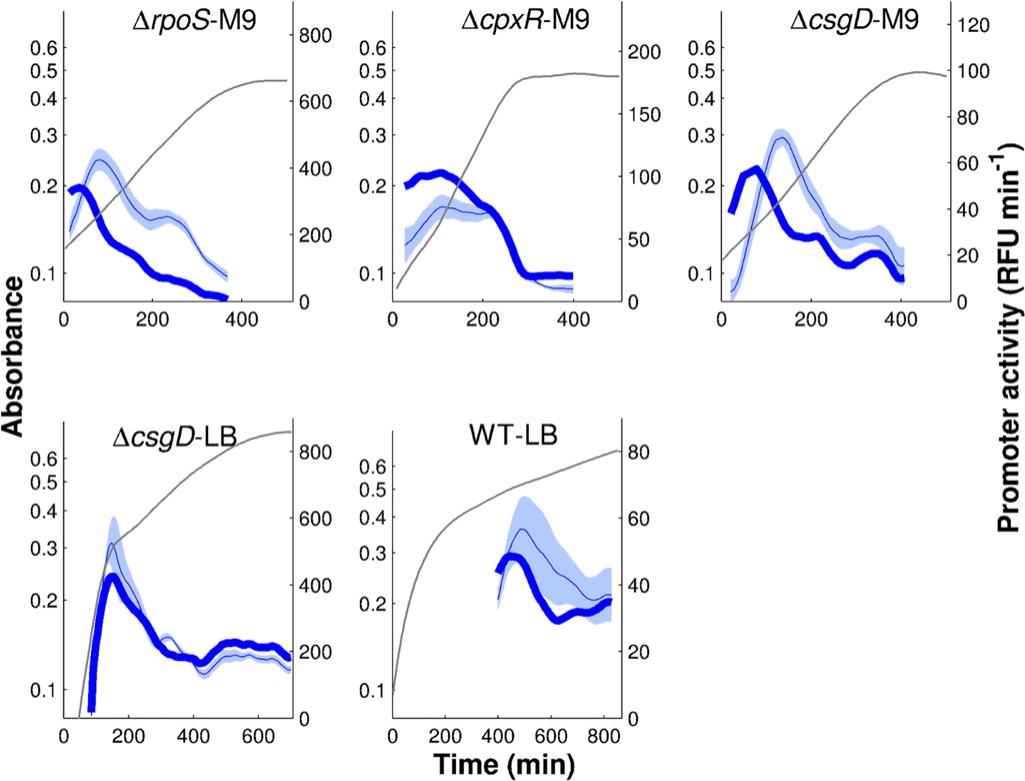
Regulation function of *tar* fitted to reporter gene data when replacing protein concentrations by promoter activities. The regulation function of Eqs. 1–2 was fitted using the promoter activities for *tar*, *fliA*, and *flgM* shown in Fig. 3, where the latter two replace the concentrations of FliA and FlgM, respectively. Model predictions are in dark blue (thick solid line), *tar* reporter data are in light blue (thin solid line and shaded area). The parameters were estimated using a multistart global optimization algorithm (see *Methods and materials* for details). The best fit returns the value *Q* = 33.4 for the objective function, for the parameter vector (*k*_0_,*k*_1_,*n*,*θ*,*K*) = (7.6,853,1,663,14615). Confidence intervals for the parameter values are reported in Text S10.

The predictions of the identified regulation function for *tar* as well as the estimated parameter values are shown in Fig. 5. We computed confidence intervals for the parameter estimates by means of a bootstrap-like procedure resampling the measured promoter activities at each time-point from an experimentally-determined distribution (see Text S10 for details). In this case, and for all parameter values reported in later figures, the confidence intervals are small (< 2-fold). This indicates that there are no identifiability issues, that is, the parameter values can be unambiguously inferred from the data [2, 7, 46].

When analyzing the estimated parameter values, we observe that the cooperativity parameter *n* equals 1 and that the equilibrium constant *K* has a value such that the regulator is fully active over the duration of the experiment (Text S9). As a consequence, the regulation function of the *tar* promoter is essentially a linear transformation of *fliA* activity. While the fit with the experimental data is quite good for the Δ*csgD*-LB and WT-LB conditions, the model is not able to account for the peak in *tar* activity in the M9 conditions. The model either predicts no peak or a peak occurring more than an hour before it is observed. In conclusion, replacing protein concentrations by promoter activities in the FliA-FlgM module is inappropriate for obtaining reliable models of the promoter activities, both structurally and quantitatively.

### Identification of gene regulation functions from promoter activities including global physiological effects

**Figure 6:**
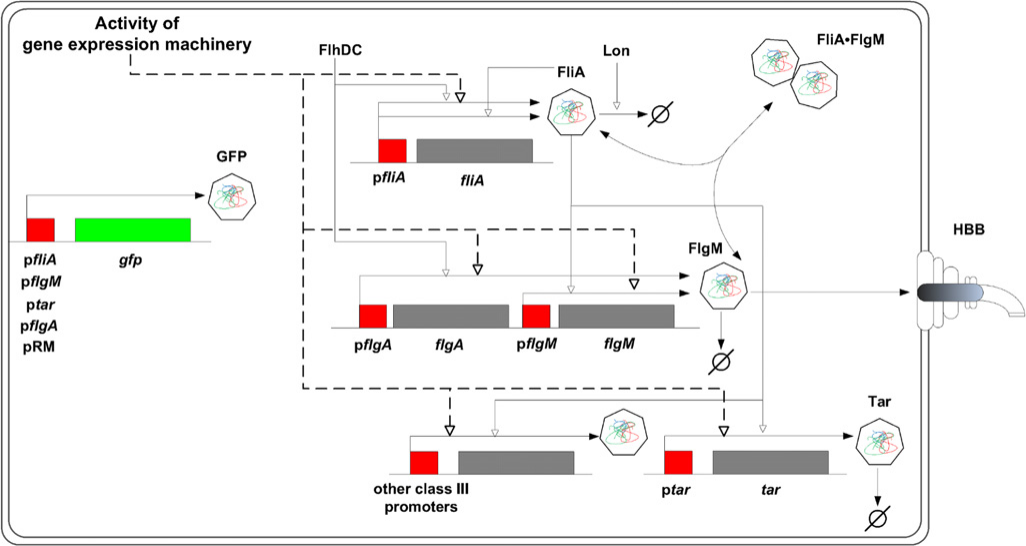
FliA-FlgM module extended with activity of the gene expression machinery. The network is the same as in Fig. 1, but the regulation of the motility genes by global physiological effects, in particular the activity of the gene expression machinery, has been included. These regulatory interactions are shown by bold, dashed lines.

A possible explanation for the difficulty to identify quantitative regulation functions from information on promoter activities alone may be that, in addition to transcription regulators and other specific regulators, the activity of the transcriptional and translational machinery also affects gene expression [16, 47–49]. Contrary to FliA and FlgM, which affect specific genes, all motility genes are affected by the activity of the gene expression machinery and other global physiological effects. Fig. 6 shows the network structure of the FliA-FlgM module when such global physiological effects are taken into account.

**Figure 7:**
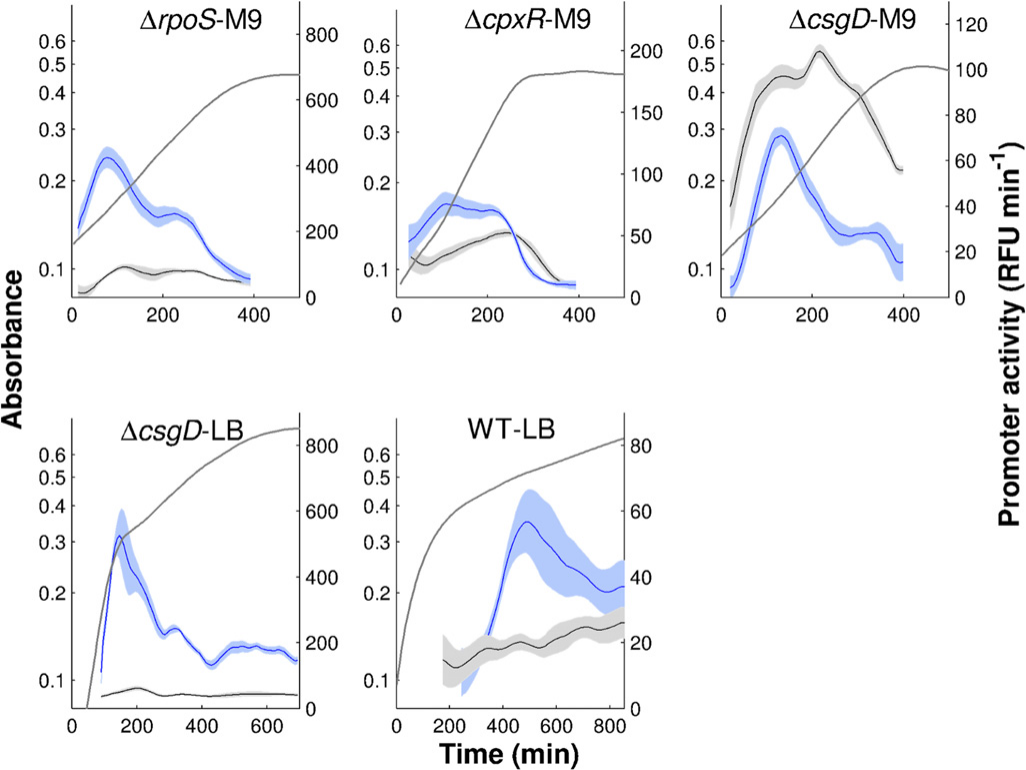
Activities of constitutive phage promoter. Activity of the phage *λ* promoter pRM (grey) and activity of *tar* (blue) measured in all experimental conditions considered in this study. The *tar* promoter activities (and the mean absorbance profiles) are the same as shown in Fig. 3.

The activity of the gene expression machinery includes the abundance and activity of RNA polymerase and ribosome, as well as pools of metabolic precursors, and is therefore difficult to quantify in a direct way. This has motivated the use of the growth rate or the activity of constitutive genes, whose expression is in principle not controlled by any specific regulators, as an indirect read-out of the global physiological state of the cell [16–17, 50]. In this study, following [17], we used the activity of the pRM promoter of phage *λ*, which is constitutive in non-infected *E. coli* cells, as a quantitative measure of the activity of the gene expression machinery and the global physiological state more generally. In Fig. 7 the time-varying activity of the constitutively-expressed reporter gene is shown, together with the activity of *tar*. Similar to the latter, in almost all conditions, the activity of the constitutive promoter shows a peak, though occurring somewhat later (in WT-LB, the detection of a peak is obscured by the analysis of fluorescence data that were extremely close to background, as witnessed by larger confidence bands).

Does the inclusion of global physiological effects enable the identification of quantitatively predictive gene regulation functions? In order to answer this question, we again applied minimal sign pattern analysis to the reporter gene data, this time including the activity of the constitutive phage promoter as a proxy for the activity of the gene expression machinery. Like in the previous section, the FliA and FlgM concentrations were replaced by the activities of their genes. The expected pattern of regulatory interactions (activation of the promoter by the gene expression machinery and FliA, inhibition by FlgM) was indeed found to be consistent with the data for *tar* (Fig. 4*B*).

We also checked if the proposed extension improves the capability of the model to quantitatively account for the time-varying activity of a FliA-controlled promoter. To this end, we multiplied Eq. 1 with *f*_*const*_(*t*), the measured activity of a constitutive promoter:

$$f\left(t\right)=f_{const}\left(t\right)\;\left[k_{0}+k_{1}\;\frac{p_{A,free}{\left(t\right)}^{n}}{\theta^{n}+p_{A,free}{\left(t\right)}^{n}}\right].$$ (3)

**Figure 8:**
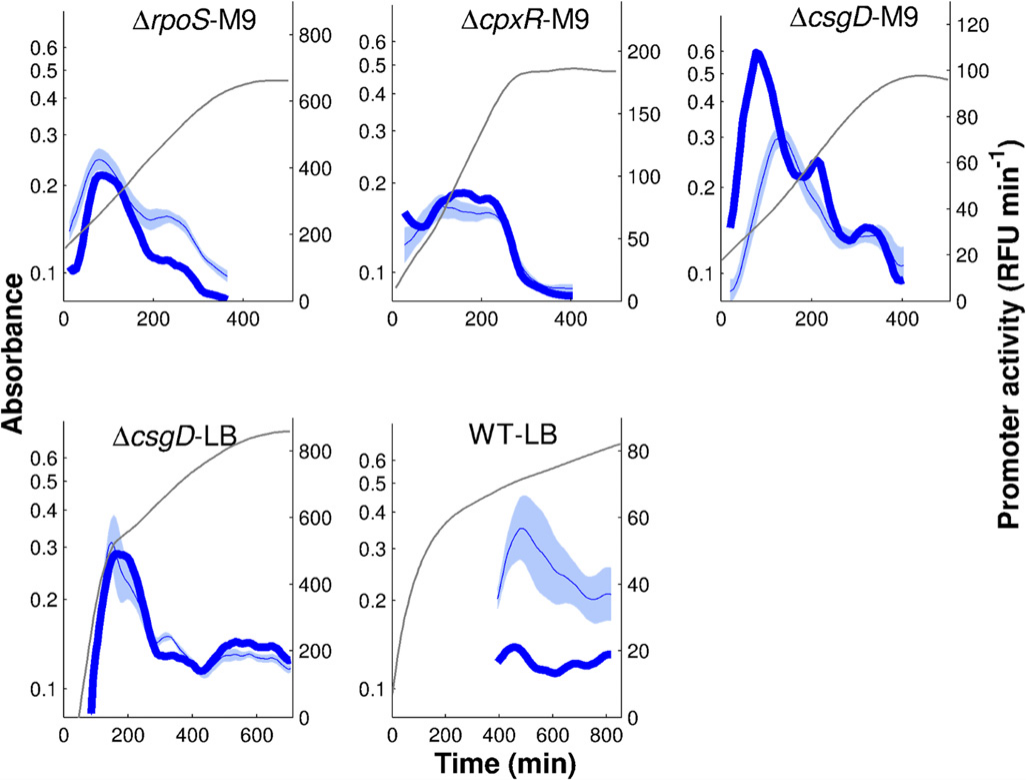
Regulation function of *tar* fitted to reporter gene data when replacing protein concentrations by promoter activities and including global physiological effects. The regulation function of Eqs. 2–3 was fitted using the promoter activities for *tar*, *fliA*, and *flgM* shown in Fig. 3, where the latter two replace the concentrations of FliA and FlgM, respectively. Moreover, global physiological effects are quantified by the activity of the constitutively expressed pRM promoter (Fig. 7). Model predictions are in dark blue (thick solid line), *tar* reporter data are in light blue (thin blue line and shaded area). The parameters were estimated using a multistart global optimization algorithm (see *Methods and materials* for details). The best fit returns the value *Q* = 30.9 for the objective function, for the parameter vector (*k*_0_,*k*_1_,*n*,*θ*,*K*) = (0.24,13.9,1.2,353,14615). Confidence intervals for the parameter values are reported in Text S10.

The fits shown in Fig. 8, obtained with the parameter estimation approach outlined in the previous section, are somewhat better than those obtained with a model accounting for the effects of FliA and FlgM only, especially for the Δ*rpoS*-M9 and Δ*cpxR*-M9 conditions. The better fit is also reflected in a lower value of the fitting error (*Q* = 30.9 vs *Q* = 33.4). Notice that the extended model has the same number of parameters as the model without global physiological effects in Eqs. 1–2, so that the improvement is not simply due to an increase in the degree of freedom of the model. The parameter estimates are quite similar to those of the previous model.

Although taking into account the activity of the gene expression machinery improves the results, the quantitative predictions of FliA-dependent regulation functions are still unsatisfactory for some conditions, notably Δ*rpoS*-M9 and WT-LB. As explained in the *Introduction*, this may be due to the use of promoter activities as proxies for protein concentrations. We therefore investigated how information on protein concentrations can be integrated into the inference process and if this improves the identification results.

### Identification of gene regulation functions from estimates of protein concentrations

**Figure 9:**
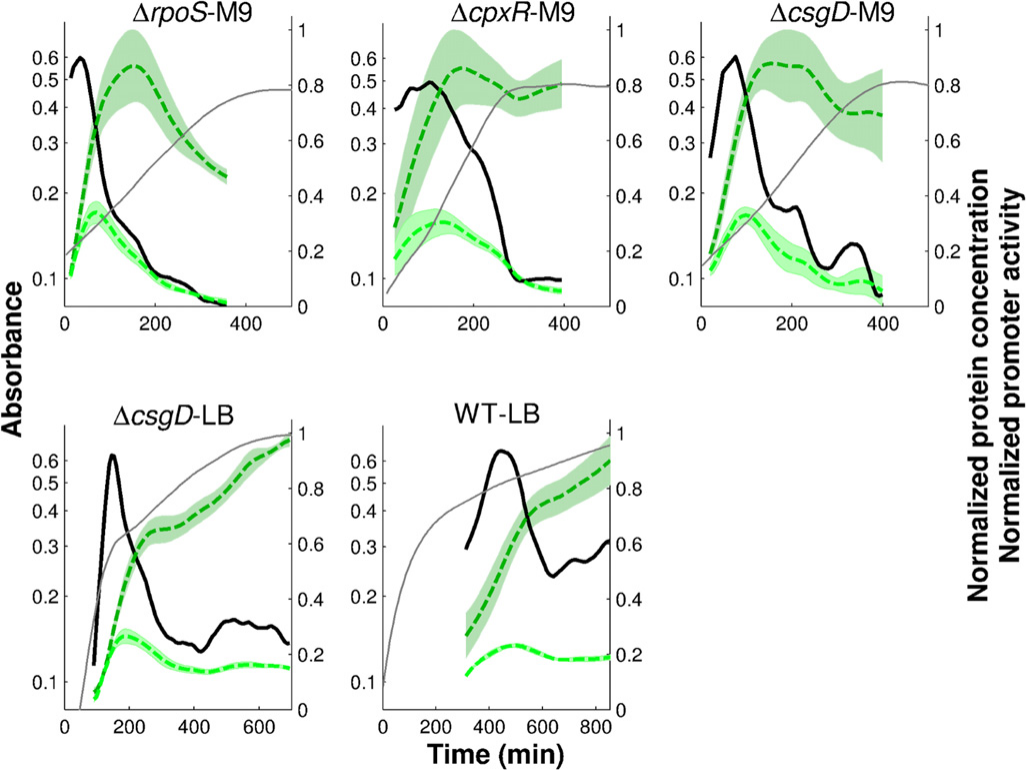
Estimates of FliA concentrations from reporter gene data. Concentrations of FliA computed from *fliA* promoter activity (thick, black solid line) in all experimental conditions considered in this study. The *fliA* activities are the same as shown in Fig. 3. The dashed, dark green line represents the concentration of the reporter protein, while the dashed, light green line represents the reconstructed FliA concentration for the measured half-live of 30 min. In each condition, promoter activity has been normalized with respect to its maximum value. The protein concentrations have been normalized with respect to the maximum of the upper limit of the confidence interval of the reporter concentration. The shaded regions correspond to the mean of the protein concentrations ± twice the standard error of the mean. For clarity, the confidence intervals of the promoter activities have been omitted.

It is straightforward to provide an estimate of the GFP concentration, by dividing the fluorescence intensity by the absorbance (*Methods and materials*). The results are shown in Fig. 9. As can be seen, the transcriptional pulse in exponential phase (Fig. 3), leading to a transient accumulation of mRNA, is seen to be followed by the prolonged presence of stable protein, indicating the temporal decorrelation of the promoter activity and the protein concentration. Unfortunately, reporter concentrations are not always representative of the concentrations of proteins of interest, that is, proteins naturally expressed from a promoter. Post-transcriptional regulation and coding bias may cause divergent synthesis rates. The main bias, however, comes from the fact that the two proteins may have different half-lives and thus different degradation rates [25].

Available data in the literature indicate that the half-lives of FliA and FlgM are much shorter than the 19 h of the GFP reporter. The measured half-lives of FliA and FlgM in *Salmonella enterica* wild-type strains growing in LB were found to be 30 min and 18 min, respectively [51]. These half-lives are much shorter than those commonly found in *E. coli*. This can be explained by the fact that, in addition to being physically degraded, FlgM is secreted from the cell (Fig. 1). Moreover, FliA is subject to active degradation by Lon [52].

How can we exploit this information to reconstruct the protein concentration from the promoter activity? As shown in [25] and the *Methods and materials* section, if the half-live of the protein of interest is known, then an estimate of its concentration can be reconstructed from the observed promoter activity using a simple kinetic model integrating the effects of protein synthesis and degradation as well as growth dilution of the protein. Fig. 9 shows the result that is obtained for the FliA concentration, using the above-mentioned half-life. Although the difference with the promoter activities is less striking than for the GFP concentrations, the computation of the concentration via integration of the corresponding activity smoothens out the rapid variations of the activities and changes the time-varying profile of the regulators.

A tacit assumption in the computation of protein concentrations from promoter activities is that the half-lives of the proteins are constant over the duration of the experiment. This may not be true in the system considered here, since the apparent half-lives of FliA and FlgM are regulated. In particular, the secretion rate of FlgM varies with the synthesis of HBB structures. Data from the literature indicate that the first FlgM molecules appear in the extracellular medium shortly after the induction of *fliA* [52, 53]. Once the cell population stops growing, the rate of assembling new flagella and thus the secretion of FlgM come to a halt as well. This increases the apparent half-lives of FliA and FlgM to 2 h and 3 h, respectively [52, 53]. Since our kinetic experiments focused on the exponential growth phase, and the analysis is limited to the time frame in which *fliA* and *flgM* are expressed, it is justified here to assume that the half-lives of FliA and FlgM are constant.

Does the estimation of time-varying protein concentrations from promoter activities, by means of a kinetic model and physiologically realistic half-lives, improve the inference of regulatory interactions and gene regulation functions?

**Figure 10:**
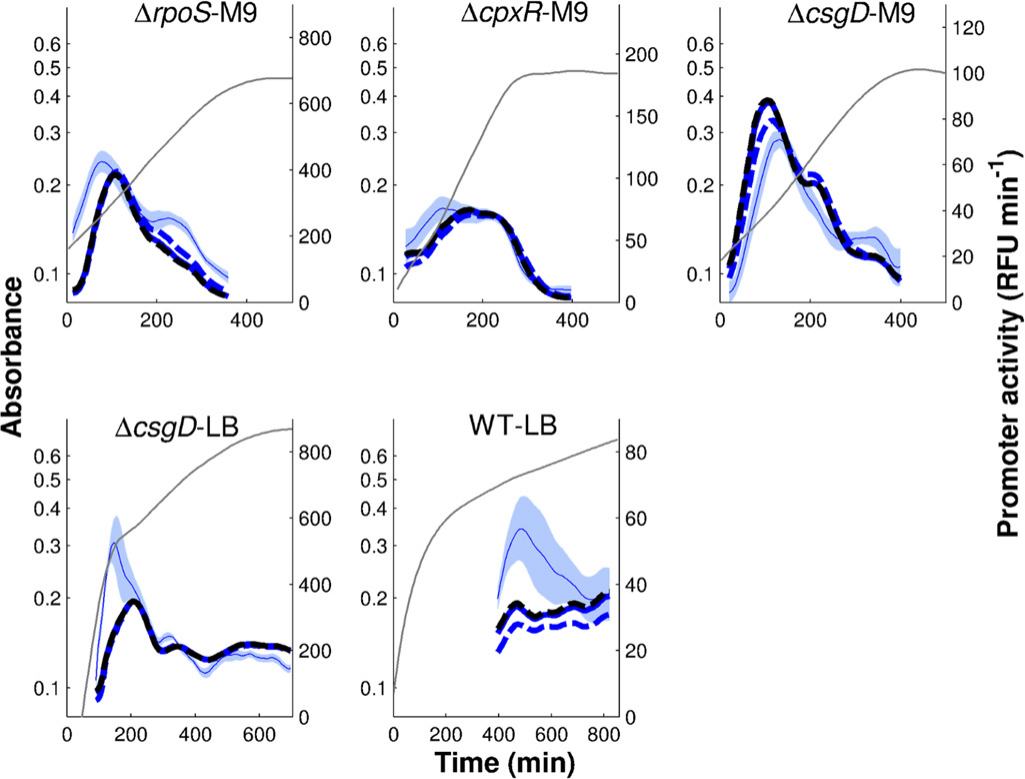
Regulation function of *tar* fitted to reporter gene data when reconstructing protein concentrations from the reporter gene data and including global physiological effects. The regulation function of Eqs. 2–3 was fit to the data using the promoter activity for *tar* (Fig. 3), concentrations of FliA and FlgM reconstructed from the activities of their promoters for physiologically realistic half-lives (Fig. 9 and Text S7), and the activity of the constitutively expressed pRM promoter quantifying global physiological effects (Fig. 7). Model predictions are in thick black and blue lines, *tar* reporter data are in light blue (thin line and shaded area). Three fits are shown: the best fit for measured half-lives of FliA and FlgM of 30 min and 18 min, respectively (thick, blue solid line, *Q* = 25.5, (*k*_0_, *k*_1_, *n*, *θ*, *K*) = (0.26, 5.0, 1.99, 3542, 447499)) and two other fits for comparable half-lives (blue and black dashed lines). Parameter values were estimated using a multistart global optimization algorithm (see *Methods and materials* for details). Their confidence intervals are reported in Text S10.

We first verified that a model using the reconstructed FliA and FlgM concentrations as regulators of *tar*, in addition to the activity of the gene expression machinery, is structurally compatible with the data. Minimal sign pattern analysis accepted the expected pattern of regulatory interactions. Second, we identified the gene regulation model of Eqs. 2–3 from the data, with the estimated FliA and FlgM concentrations for *p*_*A*_ and *p*_*M*_, respectively. As shown in Fig. 10, the model better captures the quantitative trend in the data, including in WT-LB, where the improvement was moderate though, and the resulting fit still improvable (*Q* = 25.5). Since the half-lives were taken to be those measured for a different species in growth conditions that are similar but not identical to ours, and measurement errors were not reported, we slightly relaxed the reported values. This did not much change the quality of the fit (Fig. 10). We conclude that even approximately correct half-live values may allow the results of the inference process to be improved.

The above analysis ignores a particularity of the FliA-FlgM module, namely that although the half-lives are constant in the time-window of the experiment, they may be different across growth conditions. Generally speaking, in environmental conditions favoring a larger number of flagella, and thus completed HBB structures, the secretion rate of FlgM is higher and therefore the apparent half-life shorter. For example, during growth of a wild-type strain in LB medium, the apparent half-live of FlgM is 18 min [51], but in conditions of strong induction of the flagellar hierarchy half-lives up to 7 min were measured [54]. The half-life of FliA, the flagellar sigma factor, is also variable. FliA is subject to active degradation by the Lon protease, but stabilized when bound to FlgM (Fig. 1). This makes its apparent half-life dependent on the concentration of its anti-sigma factor [52].

The observation that the half-lives of FliA and FlgM are not identical across all growth conditions considered suggests a final extension of the analysis to improve the inference results. We allowed the FliA and FlgM half-lives to vary between physiologically possible bounds in each of the conditions and estimated not only the parameters of the regulation functions, but also the half-lives. In order to reduce the computational complexity of this procedure, we discretized the possible half-live values for FliA and FlgM (27 values, between 7 min and 4 h), and we precomputed the protein concentration profiles for each half-life in each of the experimental conditions. The resulting time-course patterns were used for the same analyses as above.

**Figure 11:**
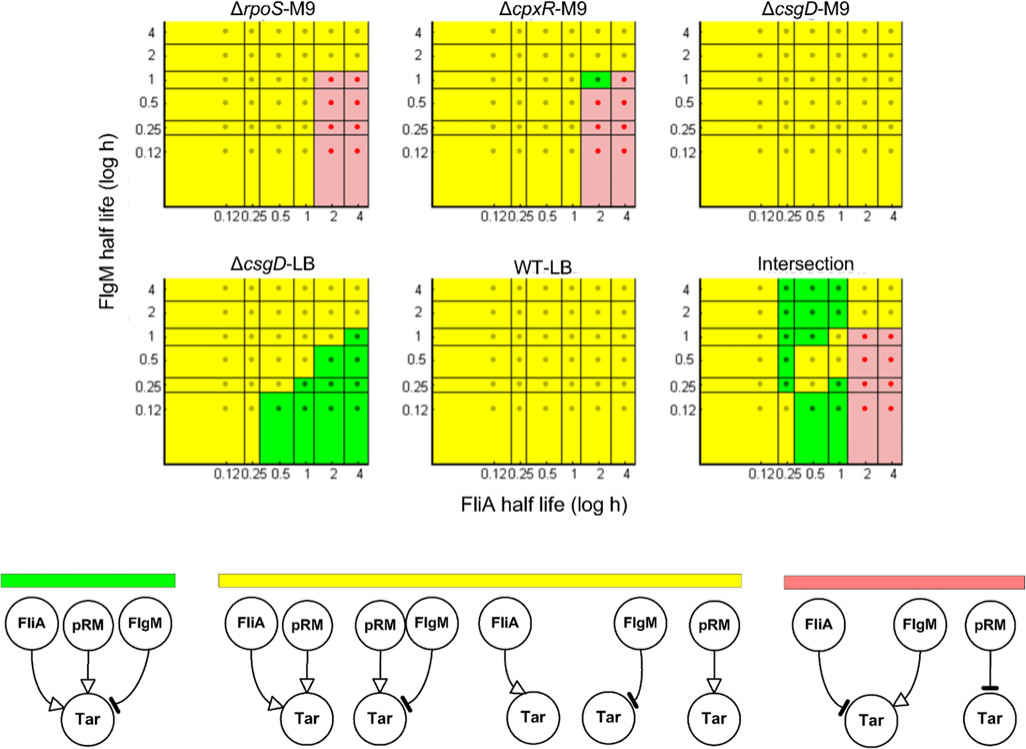
Minimal patterns of regulatory interactions for *tar* over a range of physiologically realistic half-lives. The minimal regulatory patterns for the gene *tar* in the motility network of Fig. 6 as a function of the half-lives of FliA and FlgM. The plots correspond to the five experimental conditions considered (Δ*rpoS*-M9, Δ*cpxR*-M9, Δ*csgD*-M9, Δ*csgD*-LB, and WT-LB) as well as the pooling of the data sets from all five conditions. The dot in the center of each region in the plots corresponds to a tested combination of half-lives of FliA and FlgM, and thus to specific protein concentration profiles computed from the kinetic model of gene expression (*Methods and materials*). The minimal regulatory patterns were obtained by applying the minimal sign pattern algorithm [42]. The color codes represent the different categories of minimal signal patterns inferred. A region is colored green if the expected regulatory pattern is among the minimal sign patterns returned by the algorithm, and yellow if it is compatible with the returned sign patterns. A region is colored red if none of the returned sign patterns is consistent with the data only. Two examples of inconsistent sign patterns are shown. Note that, for every combination of half-lives, the analysis of the pooled data (results reported as “Intersection”) is generally more constraining than the pooling of the results from individual analyses: The expected pattern may be consistent (yellow) with all individual datasets but not minimal (green) for any of them, and turn out to be consistent and minimal (green) when all datasets are analyzed at once (see also Text S5).

**Figure 12:**
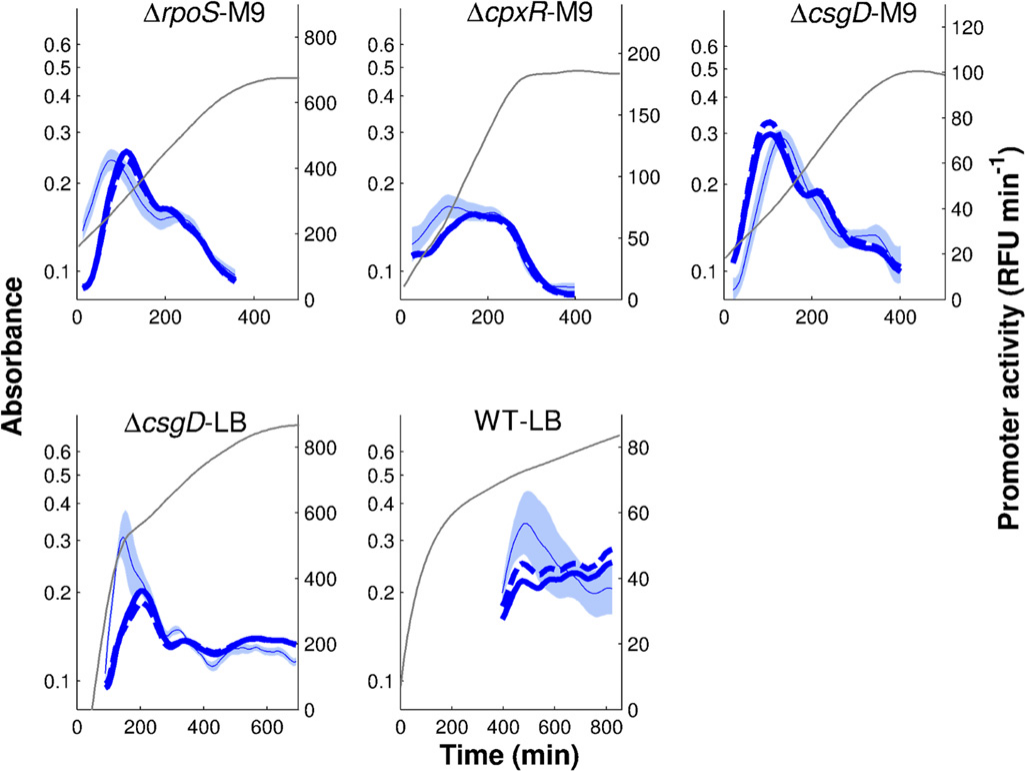
Regulation function of *tar* fitted to reporter gene data when reconstructing protein concentrations from the reporter gene data for physiologically realistic half-lives and including global physiological effects. As in Fig. 10, but the half-lives have now also been estimated from the data, within a physiologically plausible range. Model predictions are in thick solid and dashed blue lines, *tar* reporter data are in light blue (thin line and shaded area). Two example fits are shown, namely the best fit for estimated half-lives of FliA and FlgM (solid line, *Q* = 21.0, (*k*_0_,*k*_1_,*n*,*θ*,*K*) = (0.22,6.6,1.38,6252,47467)) and another example of a high-ranking fit (dashed line). In the case of the best fit, the half-lives of FliA are equal to (60,30,24,30,60) min in the (Δ*rpoS*, Δ*cpxR*, Δ*csgD*-M9, Δ*csgD*-LB, WT-LB) conditions, respectively, while the half-lives of FlgM are equal to (45,7,24,11,9) min. Confidence intervals for the parameter values are reported in Text S10.

Fig. 11 shows the results for the structural inference of *tar* regulators. As can be seen, almost all combinations of half-lives are compatible with activation of *tar* by FliA and the gene expression machinery as well as with inhibition by FlgM. This means that the returned structure of interactions is robust over the range of half-lives, a desirable property for network inference. Fig. 12 illustrates that the obtained quantitative regulation function of *tar* activity fits the data better than in all other previously considered situations (*Q* = 21.0), while the parameter values are similar to those obtained in the previous sections. Although we substantially relaxed the possible half-live values of FliA and FlgM, it is remarkable that the optimal values are close to the reported values for LB medium (Fig. 12). This emphasizes the importance of active degradation of FliA and secretion of FlgM for the dynamics of the motility network. Moreover, while the proportion of FliA released by FlgM varies across conditions, most FliA is predicted to be free over the duration of the experiment (Text S9). This is also intuitively expected, as FlgM is actively exported in the exponential growth phase considered.

We conclude that the reconstruction of protein concentrations from reporter gene data results in much better inference results for the FliA-FlgM module, for physiologically plausible values. The computation of the protein concentrations requires a simple kinetic model, accounting for protein synthesis and degradation, as well as estimates of the protein half-lives. While this increases the complexity of the data analysis procedures, it reflects the actual dynamics of gene expression and is thus critical for exploiting time-series measurements. Moreover, the availability of information on protein half-lives may not be constraining in practice, since even rough half-live estimates from the literature were seen to preserve the expected interaction pattern and provide a significant improvement of the ability of the models to quantitatively describe the time-varying promoter activity. It is important to remark, however, that adding information on protein half-lives is not enough. When repeating the identification process with the measured half-lives, but ignoring global physiological effects, the results are far worse (*Q* = 36.3, Text S7).

### Determination of conditions in which protein half-lives and global physiological effects are important

The importance of accounting for global physiological effects and protein half-lives was demonstrated above for the regulation of the expression of *tar*. The same analysis was repeated for the regulation of the *flgM* promoter. Results are reported in Text S7. We found that, for this promoter, the improvement in the fit to the experimental data obtained by including global physiological effects and protein kinetics isnot as pronounced as for *tar*. One possible explanation is that the *flgM* activity profile happens to be already well explained using the promoter activities of *fliA* and *flgM* as proxies for the corresponding protein concentrations (Text S7), thus leaving little space for improvement. In addition, from a mathematical viewpoint, we notice that using the promoter activity of *flgM* for the fitting of the same quantity may render the regression problem degenerate. Still, these results raise a more general question: When is it important to take into account protein half-lives and global physiological effects?

To answer this question we performed an *in-silico* analysis where the regulation model of Eqs. 2–3 is simulated for different protein half-lives and varying strength of the global physiological contribution, using the p*fliA*, p*flgM*, and pRM activity profiles reported in Figs. 3 and 7. Identification is then attempted from the simulated data with models ignoring protein half-lives and global physiology. This enables us to quantify the relevance of the analysis in the previous sections for a variety of realistic scenarios, starting from experimentally measured activities of bacterial promoters.

**Figure 13:**
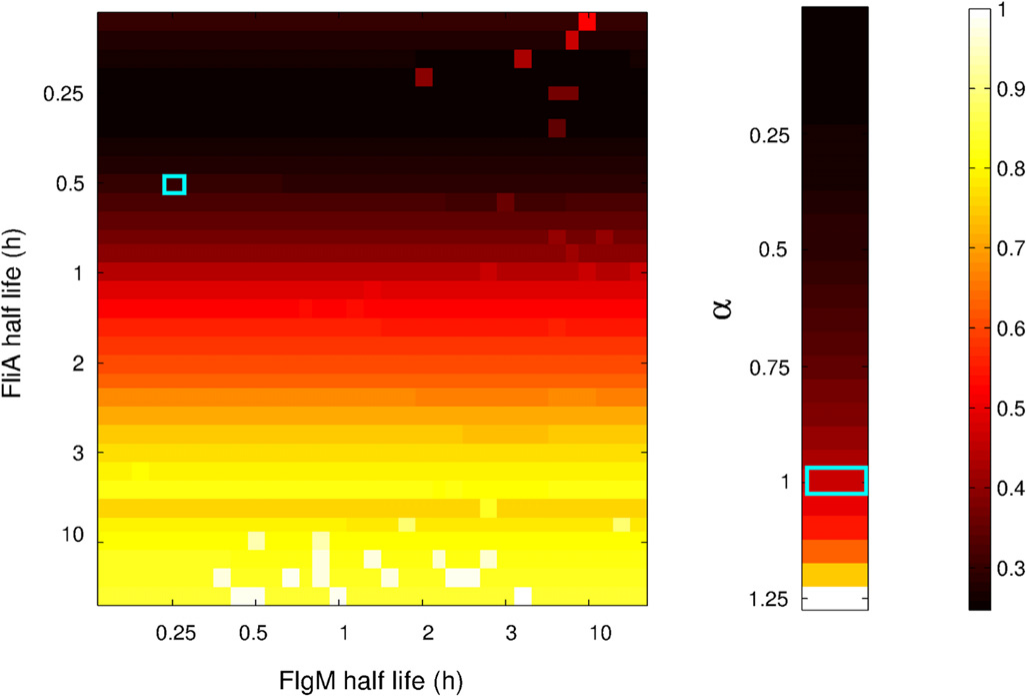
Heatmap of the fitting residuals for simulated data generated for different protein half-lives and for different strengths of global physiological effects. *A:* For all different combinations of 33 half-lives of FlgM (horizontal axis) and FliA (vertical axis), the residual of the fit for a model ignoring protein kinetics is represented by the color code reported in the right bar. For clarity of presentation, the residual values *Q* have been normalized with respect to the maximum value of *Q* over all half-life combinations. The combination corresponding to the measured half-lives in LB medium is marked with a light blue square (18 min for FlgM, 30 min for FliA). *B:* For 26 different values of the strength parameter *α*, defined in Eq. 4, the residual of the fit by a model ignoring global physiological effects is represented by the color code. The residual values *Q* have been normalized with respect to the maximum value of *Q* over the different strengths of physiological effects. The value corresponding to the real data is marked with a light blue rectangle (*α* = 1).

To evaluate the importance of protein half-lives, we simulated FliA and FlgM concentration profiles for half-lives ranging between 7 minutes and 16 hours. The other relevant parameters in the model (*k*_0_, *k*_1_, *n*, *θ*, *K*) were fixed in agreement with the best fit obtained for the reference half-lives of 30 min for FliA and 18 min for FlgM, shown in Fig. 10. More precisely, the relative position of the parameter values within the interval of physiologically plausible values, which may depend on the FliA and FlgM concentrations, as explained in the *Methods and materials*, was conserved across conditions. Activity profiles of *tar* were then generated in accordance with Eqs. 2–3 based on the experimentally measured pRM activities. We then attempted to identify from these simulated data a gene regulation model accounting for the global physiological effects, but using promoter activities in place of FliA and FlgM concentrations. The results are reported in Fig. 13.

As can be seen, the quality of the fit decreases with longer half-lives of FliA, but is rather insensitive to the half-life of FlgM. The strong dependency on the half-life of FliA shows that, in general, accounting for slow protein kinetics is important, but that promoter activities can be safely used in place of protein concentrations for very fast-degrading proteins. This is intuitively explained by the fact that fast-degrading protein concentration profiles reproduce promoter activity profiles quite closely, while this is not true in case of slow degradation (Fig. 9). The relative insensitivity to FlgM half-lives can be explained by the fact that, in the time window considered in our experimental set-up, a good fit requires most FliA to be free (Text S9). Longer half-lives, and therefore higher concentrations of FlgM, favor lower free FliA concentrations, but this tendency is compensated in the parameter optimization process by higher values for the equilibrium constant *K*. The actually measured reference half-lives of 18 min for FlgM and 30 min for FliA are located in the upper left corner of Fig. 13*A*, where fitting residuals are comparably small. Therefore, for networks involving regulators with longer half-lives than the exceptionally short half-lives observed for FliA and FlgM, it will be even more critical to account for protein kinetics than for the genes considered here.

To evaluate the importance of global physiological effects, starting from the experimentally measured pRM activity profiles, we simulated global physiological effects of different strength. In particular, we rescaled the variations of *f*_*const*_(*t*) around its temporal mean across all conditions, ${\bar{f}}_{\mathrm{const}}$, by a factor *α* ranging from 0 (no variability, no regulatory effect) to 1 (measured variability, moderate regulatory effect) and 1.25 (increased variability, strong regulatory effect). That is, synthetic activity profiles of FliA-dependent promoters were generated in accordance with the model

$$f\left(t\right)=\left[\alpha \cdot \left(f_{const}\left(t\right)-{\bar{f}}_{const}\right)+{\bar{f}}_{const}\right]\;\cdot \;\left[k_{0}+k_{1}\;\frac{p_{A,free}{\left(t\right)}^{n}}{\theta^{n}+p_{A,free}{\left(t\right)}^{n}}\right],$$ (4)

with *p*_*A*,*free*_(*t*) computed from the FliA and FlgM concentration profiles according to Eq. 2. The upper bound of 1.25 for *α* was chosen so as to avoid negative values of the promoter activity *f*(*t*).

Identification results using FliA and FlgM concentrations computed for the reference half-lives of 30 min and 18 min, respectively, but ignoring global physiological effects are reported in Fig. 13*B*. It is clear that the misfit of the *tar* promoter activity data increases with the strength of the ignored physiological effects. In particular, with the experimentally observed pRM activity (*α* = 1), the discrepancy between the data and the best model fit is quite significant. This is in agreement with the results of previous sections and especially Text S7, where it is shown that ignoring global physiological effects, even when computing protein concentrations from promoter activities, leads to poor model fits. While neglecting small variations of global physiological state (*α* ≪ 1) may be safe, ignoring highly varying global physiological effects (*α* > 1) may have even more severe repercussions on the inference results than those observed here.

In summary, the simulation study shows that, as expected, the importance of accounting for protein kinetics and global physiological effects depends on the strength of these effects, although the structure of the system itself may also play a role, as illustrated by the differences in the dependency of the fit quality on FliA and FlgM concentrations (Fig. 13*A*). As a general rule, ignoring significant fluctuations of the global physiology or large differences between mRNA and protein half-lives is very likely to result in modelling bias and hence poor inference results. Interestingly, in the previous sections a substantial improvement of the fit of a quantitative regulation function to *tar* activity was already obtained when taking into account concentrations of short-lived proteins and moderately-variable global physiological effects. In the light of the analysis of this section, the contribution of our approach becomes even more fundamental in other systems, bearing in mind that the vast majority of bacterial proteins are much more stable than FliA and FlgM, which are actively degraded and exported from the cell (Fig. 1).

## Discussion

Experimental techniques developed over the past two decades have made it possible to monitor gene expression with high precision and temporal resolution. The interpretation of these data requires reliable mathematical and computational tools for the inference of regulatory interactions as well as the identification of quantitative gene regulation functions. While enormous progress has been made on such inference methods, many problems remain. We believe that the solution of these problems should not only be sought in technical improvements of the algorithms themselves, but should also come from a better understanding of the precise information on gene expression provided by the experimental data. The relation between the primary data and physiological quantities like the cellular concentrations of mRNA and protein is usually indirect and obscured by simplifications and assumptions that do not generalize beyond the specific situations for which they were designed.

In this paper we have made explicit the relation between experimental data and physiological quantities by means of mathematical models of gene expression, calling into question two basic assumptions that are commonly made in the inference of regulatory interactions and quantitative gene regulation functions from time-series data.

The first assumption is that transcriptome data alone are sufficient to capture the time-varying state of gene expression. Often, the regulators of gene expression are proteins and, whereas mRNA and protein concentrations are correlated at steady state, this is generally not the case when the two are considered dynamically over time. As a consequence, neglecting the distinction between mRNA and protein may hamper the full and correct exploitation of the information contained in time-series transcriptome data. This might explain why the comprehensive evaluation of network inference methods carried out in the DREAM initiative concluded that steady-state transcriptome data comparing wild-type and mutant strains are usually more informative for network inference than time-series data [55]. The temporal decorrelation of the mRNA and protein concentrations makes the former generally an unreliable proxy of the latter.

A second implicit assumption in the analysis of transcriptome data is that gene regulation can be reduced to the action of transcription factors and other specific regulators. This ignores the fact that the activity of the transcriptional and translational machinery, as well as other global physiological effects such as gene copy number and DNA supercoiling, may drastically change over the course of an experiment, a fact that has been well-documented for microorganisms [56–58]. As Lovén *et al*. demonstrate, a global increase or decrease of transcriptional activity across conditions may lead to erroneous interpretations and the inference of spurious regulatory interactions [21].

The main contribution of this paper is an integrated experimental and computational approach for addressing the above two problems, in the context of time-series measurements of gene expression by means of fluorescent reporter genes. We propose new controls for transcriptome experiments, in particular the use of constitutively-expressed genes, as well as mathematical models and computational procedures for reconstructing protein concentrations and for integrating global physiological effects into the network inference process. The reconstruction of protein concentrations from real-time promoter activities by means of kinetic models as well as the quantification of global physiological effects by means of reporter genes have been proposed before [17–18, 25, 59]. For instance, Gerosa *et al*. have developed quantitative models to dissect global and specific regulation of *E. coli* genes involved in arginine biosynthesis [18]. To our knowledge, however, the work presented here is the first systematic study of how the integration of information on both global physiological effects and protein concentrations can improve the inference of regulatory interactions and the identification of regulation functions from time-series gene expression data.

It is important to emphasize that the proposed approach is orthogonal to existing inference methods and that the models and analysis procedures proposed in this study can be directly combined with many of the methods described in the literature [1, 3–7]. The models and analysis procedures we have used are explicitly detailed and can be easily integrated into available methods, as illustrated for the Matlab implementation of the minimal sign pattern algorithm [42]. While reporter gene data were used in this study, other experimental techniques may also yield time-series transcriptome data suitable for our purpose. The main requirement is that an estimate of the expression of a constitutive gene can be obtained and sampling times are sufficiently dense and precise to allow time-varying mRNA concentrations to be reliably measured.

We have validated our approach by means of a central module of the motility network in *E. coli*. The FliA-FlgM module has been very well-studied and has characteristics that make it atypical but particularly suitable for our purpose. FlgM is secreted from the cell and FliA is a target for proteolysis, which causes these regulators to have apparent half-lives that are quite short in comparison with typical *E. coli* proteins. Moreover, the secretion and degradation rates may change across conditions, depending on the strength of induction of the flagella synthesis network. This yields a rich and challenging data set for testing how accounting for the distinction between cellular responses on the level of mRNA and protein influences the results of the inference process.

We investigated the capability to infer from reporter gene data both the regulatory structure and the quantitative regulation function of a FliA-dependent motility gene, not known to be regulated by any other transcription factors. When progressively solving the problems mentioned above, by integrating information on the activity of the gene expression machinery and computing estimates of protein concentrations from promoter activities, both the structure and the dynamics of the regulation of the *tar* promoter could be identified successfully. We emphasize that, when using available measurements of FliA and FlgM half-lives, this was achieved without increasing the number of parameters in the models and is therefore not simply a consequence of increasing the degrees of freedom. Moreover, *a-posteriori* analysis of the confidence intervals of the parameter estimates (Text S10) confirmed that there are no identifiability issues, that is, the models are fully determined by the available data.

The results underline the important roles played by global physiological effects and the active regulation of FliA and FlgM half-lives in shaping the dynamics of FliA-dependent promoters. When global physiological effects were ignored, or the FliA and FlgM half-lives were set to typical values of *E. coli* proteins, a sharp drop in the quantitative predictivity of the gene regulation models was observed (Text S7). In other words, both the inclusion of global physiological effects and realistic half-lives were necessary to improve the inference results in our example network.

More generally, under which conditions does the inclusion of the above factors lead to better results and when can they be ignored? We performed a simulation study in which we systematically varied the relative contribution of global physiological effects to cross-condition variations in the expression of a target gene and the half-lives of the regulators. These results showed that longer half-lives of the activating transcription factor and stronger variations of global physiological effects make it more difficult to obtain good fits when using promoter activities and data on specific regulators only, respectively. While these conclusions are not surprising, it is important to emphasize that in the system studied here, where FliA and FlgM have half-lives that are exceptionally short for bacterial proteins, a considerable improvement of the fit could be obtained. For regulatory proteins with more typical half-lives, the gain may therefore be even more important than observed here.

The proposed approach to better exploit the information contained in time-series data of the trancriptional response of bacterial cells depends on kinetic models of gene expression, relating the primary fluorescence and absorbance data to promoter activities and protein concentrations. The models used in this study could be further refined, by taking into account delays that are due to the maturation of GFP and the time for rounds of transcription and translation to complete [25, 60–62]. These refinements were neglected here, since the GFP reporter used in this study is fast-folding and the transcription and translation delays are short on the time-scale of the experiments. The computation of protein concentrations by means of these measurement models depends on the availability of approximate values of the protein half-lives. While genome-wide studies of the stability of individual proteins exist, *e.g.*, for yeast [63], quantitative information on the stability of individual proteins in microorganisms is seldom available. Still, it is known that most proteins in *E. coli* are stable, with half-lives >10 h. In most experimental scenarios in the laboratory, bacterial growth occurs at a much higher pace, *i.e.*, *μ* ≫ *γ*_*p*_, which is sufficient to ensure correct applicability of our measurement models regardless of the specific (poorly known) value of *γ*_*p*_. Turned another way, to apply our measurement models in cases of poorly known half-lives of stable proteins, it suffices to perform experiments with cell doubling times well below 10 h.

In conclusion, the applicability of our principled approach to account for protein degradation and global effects in network reconstruction from reporter gene data goes well beyond the simple and well-understood biological system on which it was illustrated and the specific network analysis and identification methods utilized. In fact, the use of the proposed approach becomes even more important in problems involving networks that are less known and/or of greater complexity, in that the identification problem becomes intrinsically more difficult, and therefore the biases introduced by common though weakly justified hypotheses or approximations become even more difficult to discern. Due to the generality of both the problem and the proposed solutions, we believe that the methodology presented in this paper has broad practical applicability for analyzing time-series transcriptome data and improving network inference in a variety of organisms.

## Methods and materials

### Strains and growth conditions

The *E. coli* strains used for this study are the wild-type strain BW25113 and isogenic deletion mutants Δ*rpoS*, Δ*csgD* and Δ*cpxR*. The strains were taken from the Keio collection [64] and the kanamycin resistance cassette was removed [40]. The wild-type and mutant strains were transformed with low-copy plasmids bearing a fusion of a *gfp*mut2 reporter gene with the promoter regions of the genes *tar*, *fliA*, and *flgM*. These plasmids were selected from the plasmid library constructed at the Weizmann Institute [65]. A reporter for the pRM promoter of phage *λ* was constructed in the same plasmid vector to provide information on the physiological state of the bacteria, following the approach in [17]. The pRM promoter fused with the *gfp* reporter gene was also inserted into the chromosome of the BW25113 wild-type strain as reference for the qRT-PCR assays. All plasmids carry the kanamycin resistance gene. All the strains and plasmids were verified by PCR. More details on the strains and plasmids used in this study can be found in Text S1.

The strains were recovered from glycerol stock and grown overnight (16 h) at 37°C in LB rich medium and M9 minimal medium [66] supplemented with 0.3% glucose and mineral trace elements. For the preculture of strains containing plasmids, kanamycin (50 *μ*g/ml) was added. The overnight cultures were diluted (10- to 100-fold) into a 96-well microplate, so as to obtain an adjusted initial OD_600_ of 0.2. The wells of the microplate contain 150 *μ*l of the above medium, to which was added 1.2% of the buffering agent HEPES (4-(2-hydroxyethyl)-1-piperazineethanesulfonic acid) for maintaining a constant external pH. The wells were covered with 60 *μ*l of mineral oil to avoid evaporation. The microplate cultures were then grown for about 16 h at 37°C, with agitation at regular intervals, in a microplate reader (Fusion Alpha, Perkin-Elmer).

### Experimental monitoring of gene expression in real time

The expression of the fluorescent reporter genes in different genetic backgrounds and different growth media was monitored *in vivo* and in real time. About 150 readings each of absorbance (600 nm) and fluorescence (485/520 nm) were obtained during a typical experiment using the Perkin-Elmer microplate reader. In order to compute promoter activities and protein concentrations from these data, data analysis procedures were designed and implemented in Matlab, completing earlier work [17, 25]. These data procedures account for the specific half-life of the fluorescent reporter protein and take special care in the subtraction of the autofluorescence background (see Text S3 for details on the data analysis procedures).

Denoting by *A*(*t*) and *I*(*t*) the (background-corrected) time-varying absorbance and fluorescence signals, we computed the reporter concentration *r*(*t*) and the promoter activity *f*(*t*) by means of the following formulas:

$$r(t)\;=\frac{I(t)}{A(t)},$$ (5)

$$f(t)\;=\frac{d}{dt}r(t)+(\gamma_{r}+\mu (t))r(t)=\frac{\frac{d}{dt}I(t)}{A(t)}+\gamma_{r}\frac{I(t)}{A(t)},$$ (6)

where *γ*_*r*_ [min^−1^] is the degradation constant of the reporter and *μ*(*t*) [min^−1^] the growth rate of the bacteria. The half-life of the protein is defined as *t*_1/2_ = ln2/*γ*_*r*_. The reporter concentration is expressed in units RFU and the promoter activity in units RFU min^−1^, as is usual for this kind of measurements (see [17] and Text S3). The growth rate is easily estimated from the time-varying absorbance, using the standard relation *μ*(*t*) = *d* ln*A*(*t*)/*dt*. The above equations rely on the use of a kinetic model of the expression of the reporter gene, as explained in Text S2. We used cubic smoothing splines (csaps function in Matlab) to fit the fluorescence and absorbance data and obtain estimates of *A*(*t*), *I*(*t*), *dA*(*t*)/*dt*, and *dI*(*t*)/*dt*. The half-life of the GFPmut2 reporter is 19 h (*γ*_*r*_ = 0.0006±0.0001)). The maturation time of GFPmut2 is so short (4 min, [65]) that it can be safely ignored.

A similar measurement model was used for the expression of the actual gene of interest, encoding a protein with concentration *p*(*t*) [RFU min^−1^]:

$$\frac{d}{dt}p\left(t\right)=f\left(t\right)-\left(\gamma_{p}+\mu \left(t\right)\right)\;p\left(t\right),\;\;\;p\left(0\right)=p_{0},$$ (7)

where *γ*_*p*_ [min^−1^] is the degradation constant of the protein. Notice that in the case of FlgM, protein degradation includes both physical degradation of the protein and secretion through the cell membrane. When the degradation constant is known, we can compute the protein concentration by numerical integration, starting from the initial concentration *p*_0_. This initial concentration is obtained from the reporter gene data, by realizing that the bacterial cells at the beginning of the experiment are rediluted cells from a preculture grown in the same medium. In particular, assuming that gene expression in the preculture is at steady-state, it follows from Eq. 7 that

$$p(0)=p(T)=\frac{\mu (T)+\gamma_{r}}{\mu (T)+\gamma_{p}}r(T),$$ (8)

where *μ*(*T*) is the growth rate at the end of the preculture (at time *T*), *p*(*T*) and *r*(*T*) are the corresponding concentrations of the protein of interest and reporter protein, respectively. Usually, the bacteria in the preculture are in stationary phase, so *μ*(*T*) = 0. Eq. 8 was solved by numerical integration using the quad function in Matlab.

In the case of the motility network there are two complications that slightly modify this general scheme. First, the half-lives of FliA and FlgM are variable over the time-course of the experiment. During exponential growth, when the motility genes are expressed, FliA and FlgM have short half-lives, due to proteolysis and secretion, respectively. During stationary phase, at the end of the preculture, this is no longer the case and FliA and FlgM have longer half-lives (2 h for FliA and 3 h for FlgM [52, 53]). As a consequence, when computing the initial protein concentrations from the reporter concentrations at time *T*, we need to take protein degradation constants ${\gamma^{\prime }}_{p}$ corresponding to these longer half-lives. Second, in some experimental conditions, notably in rich medium like LB, the activity of the *fliA*, *flgM*, and *tar* promoters is negligible in the first few hours of the experiment [27]. As a consequence, the fluorescence intensity in the corresponding reporter strains is indistinguishable from the background fluorescence. We assume the promoter activity of the genes to be 0 in this case and back-extrapolate the observed promoter activities at earlier times towards 0. In Text S8 we illustrate the effects of variable half-lives and extrapolation of promoter activities on the computation of FliA and FlgM concentrations in a WT strain grown in LB.

For each of the derived quantities *r*(*t*), *f*(*t*), and *p*(*t*), confidence intervals (defined as ±2 standard errors of the mean) were computed from 6–7 experimental replicates.

### Relative quantification of gene expression using real-time qRT-PCR

We verified the reporter gene measurements by means of qRT-PCR in the WT-LB condition, following a previously validated protocol [67]. Details of the experimental procedure can be found in Text S6.

### Inference of minimal patterns of regulatory interactions

We use the method introduced in [42] to infer minimal pattern of regulatory interactions from time-series reporter gene data. The assumption of the method is that a regulator (*e.g.*, a transcription factor, but also the gene expression machinery) cannot operate both as a repressor and as an activator of a target gene, while it is allowed to operate as a repressor for one gene and as an activator for another gene. This corresponds to assuming that the activity of a gene is a monotone nondecreasing function of activators and a monotone nonincreasing function of repressors. Any such regulatory pattern can be encoded in terms of a sign pattern, *i.e.*, a vector containing one entry per regulator, taking value +1 for activators, −1 for repressors, and 0 for factors that do not affect the expression of the gene under consideration.

For every target gene, the method scans the measured promoter activities and concentrations of putative regulators. A sign pattern, *i.e.* a hypothesis on the regulatory structure, is rejected if it is found to be inconsistent with the data, *i.e.*, if measurements violate the monotonicity properties corresponding to that sign pattern. For instance, in the network module considered in this paper, the assumption that both FlgM and FliA activate *tar* can be rejected if any two measurement times are found such that, for higher concentrations of FlgM and FliA, the promoter activity of *tar* is lower. The algorithm makes the above verifications in a computationally efficient way and returns, for every target gene, a set of minimal sign patterns. The minimal sign patterns are regulatory patterns consistent with the data, having the properties that removal of any interaction results in an inconsistent pattern, whereas addition of a regulator (activator or repressor) preserves the consistency. In order to check the robustness of the minimal patterns thus obtained, we verified that no sign patterns were dismissed because of a single pair of measurements in the time-series. Mathematical details on the minimal sign pattern method can be found in Text S5 and [42].

### Parameter estimation

The promoter activity models we considered in the main text have the form f(t)=f(x(t),c), where *c* is a vector of unknown parameters and *x* is a vector of regressors. The specific form of f(x(t),c) is given in Eq. 1 and 3. The regressors take different forms in consecutive sections of this paper, consisting either of the activities *f*_*A*_ and *f*_*M*_ of the *fliA* and *flgM* promoters (*x* = (*f*_*A*_,*f*_*M*_)) or the reconstructed concentrations *p*_*A*_ and *p*_*M*_ of FliA and FlgM (*x* = (*p*_*A*_,*p*_*M*_)). In all sections, *c* = (*k*_0_,*k*_1_,*n*,*θ*,*K*), as defined in the section *Identification of gene regulation functions from promoter activities*. The superscript symbol *s* indicates the experimental condition, where s∈S={ΔrpoS-M9,ΔcpxR-M9,ΔcsgD-M9,ΔcsgD-LB,WT-LB}. Given measurements $\left({\bar{x}}^{s}(t),{\bar{f}}^{s}(t)\right)$ of (x(t),f(t)) (averages of 6–7 experimental replicates) at times $t\in T^{s}$ along with confidence intervals (${\bar{f}}^{s}(t)\pm \epsilon^{s}(t)$) (computed from the same experimental replicates with *ε* ^*s*^ equal to twice the standard error of the mean ${\bar{f}}^{s}$), we estimate *c* by solving the optimization problem

$$\hat{c}=\min_{c\in C}Q(c),\;Q(c)=\sum_{s\in S}\sum_{t\in T^{s}}\frac{1}{2\in^{s}(t)}\left|{\bar{f}}^{s}(t)-f({\bar{x}}^{s}(t),c)\right|.$$

The solution is found in Matlab using the multistart global search function gs with standard settings (interior-point method, fmincon for local minimizations). We tried several other global optimization function available in the Matlab global search toolbox, but the gs function was found to perform best. The parameter search space *C* is given by the constraints

$$0\leq k_{0}\leq \max\{{\bar{f}}^{s}(t):\;t\in T^{s},s\in S\},$$

$$0\leq k_{1}\leq 10\cdot \max\{{\bar{f}}^{s}(t):\;t\in T^{s},s\in S\},$$

1≤n≤4,

$$0\leq \theta \leq \max\{{\bar{x}}_{1}^{s}(t):\;t\in T^{s},s\in S\},$$

$$0\leq K\leq 20\cdot \max\{{\bar{x}}_{2}^{s}(t):\;t\in T^{s},s\in S\}.$$

The above procedure applies to the estimation of the regulation function of both *tar* and *flgM*. However, for the estimation of the regulation function of *flgM*, the condition WT-LB is not available and hence excluded from the computation of *Q*(*c*). Moreover, in the latter case, *K* is fixed for biological consistency to the value inferred from the fitting of *tar* promoter activity. For all parameter fits shown in the main text, we performed *a-posteriori* identifiability analysis to ensure that no structural or practical identifiability issue affects our results (Text S10). Overfitting issues were also excluded based on the results of this analysis and visual inspection of the fits.

## Supporting Information

S1 Text Strains and plasmids. (PDF)

S2 Text Measurement models for reporter gene data. (PDF)

S3 Text Data analysis. (PDF)

S4 Text Derivation of regulation function of motility genes. (PDF)

S5 Text Computation of minimal consistent sign patterns. (PDF)

S6 Text Validation of reporter gene data using qRT-PCR. (PDF)

S7 Text Additional data, analysis results, and fits. (PDF)

S8 Text Initial conditions for computing protein concentrations. (PDF)

S9 Text Parameter analysis: computation of active FliA. (PDF)

S10 Text Computation of parameter confidence intervals. (PDF)
