# Correction: Inference of Quantitative Models of Bacterial Promoters from Time-Series Reporter Gene Data

**DOI:** 10.1371/journal.pcbi.1005402

**Published:** 2017-03-08

**Authors:** The PLOS Computational Biology Staff

## Notice of Republication

This article was republished on May 5th, 2015, to correct errors in the figures that were introduced during the typesetting process. The publisher apologizes for the errors. Please download this article again to view the correct version.
