# Supplementary material for: Inference of Quantitative Models of Bacterial Promoters from Time-Series Reporter Gene Data
Source: PLoS Comput Biol. 2015 Jan 15;11(1):e1004028. doi: 10.1371/journal.pcbi.1004028 (PMC4295839; doi:10.1371/journal.pcbi.1004028)
Supplement: S1 Text — (PDF) [file pcbi.1004028.s001.pdf]

Supplementary Text S1: Strains and plasmids[a]

Diana Stefan[1,2], Corinne Pinel[1,2], Stéphane Pinhal[1,2], Eugenio Cinquemani[1], Johannes Geiselmann[1,2], Hidde de Jong[1,*]
1 INRIA Grenoble – Rhône-Alpes, Grenoble, France
2 Laboratoire Interdisciplinaire de Physique (LIPhy, CNRS UMR 5588), Université Joseph Fourier, Grenoble, France
∗ Corresponding author. INRIA Grenoble—Rhône-Alpes, 655 avenue de l'Europe, Monbonnot, 38334 Saint-Ismier Cedex, France. Tel.: +33476615335, Fax: +33456527120, Email: Hidde.de-Jong@inria.fr

The *E. coli* strains used in this study are all derived from the wild-type strain BW25113.In particular, we used the $\Delta rpoS$, $\Delta cpxR$ and $\Delta csgD$ deletion strains of BW25113 taken from the Keio collection [1]. The mutants were reconstructed in our laboratory [2] in order to eliminate the kanamycin resistance gene present in the original deletion strains (Table 1 in this text).

Strain	Characteristics	Reference or source
WT	*E. coli* BW25113	[1]
WTpRM	*E. coli* BW25113 pRM-*gfp*::*intS*	This study
$\Delta rpoS$	*E. coli* BW25113 $\Delta rpoS$	[2]
$\Delta cpxR$	*E. coli* BW25113 $\Delta cpxR$	[2]
$\Delta csgD$	*E. coli* BW25113 $\Delta csgD$	[2]

Table 1. Strains used in this study.

The wild-type and mutant strains were transformed with plasmids bearing a *gfp* reporter gene, encoding the stable GFPmut2 reporter (Table 2 in this text). The reporter plasmids for the genes *tar*, *fliA*, *flgM*, and *flgA* were selected from the plasmid library developed at the Weizmann Institute [3]. These low-copy pUA66*gfp* plasmids are kanamycin-resistant and have the origin of replication of the pSC101 plasmid. The reporter plasmids are transcriptional fusions of the promoter regions of the genes of interest and the *gfp* sequence. The same vector was used to construct a reporter for the constitutive promoter pRM of λ phage, by cloning the pRM promoter region from the pZE1RM*gfp* plasmid used in [4] into the pUA66*gfp* plasmid backbone. Table 3 in this text lists the primer sequences used for the construction of this pUA66pRM-*gfp* plasmid. The above-mentioned plasmids were transformed into the wild-type and deletion strains of Table 2 in this text. We verified that the plasmids do not modify the growth of the transformed strains. All strains and plasmids were verified by PCR.

The pRM promoter fused with the *gfp* reporter gene was also inserted into the chromosome of the BW25113 wild-type strain as reference for the qRT-PCR assays. The WTpRM strain was constructed according to the linear DNA recombination protocole of Sharan *et al.* [5]. The pRM promoter region along with the gene encoding the GFPmut3 reporter were introduced into the *intS* locus on the chromosome of BW25113 WT strain, by means of the λ Red system. The pRM-*gfpmut3* sequence was recovered from the pZE1RM*gfp* plasmid used in [4]. The recombineering protocols use the bacteriophage λ Red system that includes the phage recombination genes *gam*, *bet* and *exo*. The protein encoded by *gam*, Gam, prevents *E. coli* nuclease from degrading linear DNA fragments [6, 7] thus allowing preservation of transformed linear DNA *in vivo*. The *bet* gene product, Beta, is a ssDNA binding protein that promotes annealing of two complementary DNA molecules [8], and the *exo* gene product, Exo, has a 5 to 3dsDNA exonuclease

[a]This text contains supplementary information for the paper "Inference of quantitative models of bacterial promoters from time-series reporter gene data".

activity [9]. Working together these latter two proteins insert linear DNA at the desired target, creating genetic recombinants.

Plasmid	Characteristics	Reference or source
pUA66*gfp*	Kan^r, pSC101 *o*ri, *g*fpmut2	[3]
pUA66*fliA-gfp*	Kan^r, pSC101 *ori*, *fliA-gfpmut2*	[3]
pUA66*flgM-gfp*	Kan^r, pSC101 *ori*, *flgM-gfpmut2*	[3]
pUA66*flgA-gfp*	Kan^r, pSC101 *ori*, *flgA-gfpmut2*	[3]
pUA66*tar-gfp*	Kan^r, pSC101 *ori*, *tar-gfpmut2*	[3]
pUA66pRM-*gfp*	Kan^r, pSC101 *ori*, p*RM-gfpmut2*	This study

Table 2. Plasmids used in this study.

Plasmid	Primer sequence
pUA66pRM-*gfp*	pRM-fw: GAGGC CCTTT CGTCT TCACC TCGAG CCTAT CACCG CCAGA
	pRM-re: TTCTT AAATC TAGAG GATCC GGTTT CTTTT TTGTG CTGAT
	gfp-fw: ATCAG CACAA AAAAG AAACC GGATC CTCTA GATTT AAGAA
	gfp-re: TCTGG CGGTG ATAGG CTCGA GGTGA AGACG AAAGG GCCTC

Table 3. Primers used for the construction of pUA66pRM-*gfp* plasmid. The pUA66pRM-*gfp* plasmid was constructed with the Gibson Assembly method [10]. The pUA66*gfp* plasmid backbone was amplified using the primers gfp-fw and gfp-re. The pRM promoter region was amplified from the pZE1RM*gfp* plasmid [4] using the primers pRM-fw and pRM-re. pRM-fw and pRM-re contain the XhoI and BamHI restriction sites, respectively.

References

1. Baba T, Ara T, Hasegawa M, Takai Y, Okumura Y, et al. (2006) Construction of *Escherichia coli* K-12 in-frame, single-gene knockout mutants: the Keio collection. Mol Syst Biol 2: 2006.0008.

2. Dudin O, Geiselmann J, Oqasawara H, Ishihama A, Lacour S (2014) Repression of flagellar genes in exponential phase by CsgD and CpxR, two crucial modulators of *Escherichia coli* biofilm formation. J Bacteriol 196: 707-15.

3. Zaslaver A, Bren A, Ronen M, Itzkovitz S, Kikoin I, et al. (2006) A comprehensive library of fluorescent transcriptional reporters for *Escherichia coli*. Nat Methods 3: 623-8.

4. Berthoumieux S, de Jong H, Baptist G, Pinel C, Ranquet C, et al. (2013) Shared control of gene expression in bacteria by transcription factors and global physiology of the cell. Mol Syst Biol 9: 634.

5. Sharan S, Thomason L, Kuznetsov S, Court D (2009) Recombineering: a homologous recombination-based method of genetic engineering. Nat Protocols 4: 206-23.

6. Karu A, Sakaki Y, Echols H, Linn S (1975) The gamma protein specified by bacteriophage lambda. Structure and inhibitory activity for the RecBC enzyme of *Escherichia coli*. J Biol Chem 250: 7377-87.

7. Murphy K (1991) Lambda Gam protein inhibits the helicase and chi-stimulated recombination activities of *Escherichia coli* RecBCD enzyme. J Bacteriol 173: 5808-21.

8. Karakousis G, Ye N, Li Z, Chiu S, Reddy G, et al. (1998) The beta protein of phage lambda binds preferentially to an intermediate in DNA renaturation. J Mol Biol 276: 721-31.

9. Cassuto E, Lash T, Sriprakash K, Radding C (1971) Role of exonuclease and beta protein of phage lambda in genetic recombination. V. Recombination of lambda DNA *in vitro*. Proc Natl Acad Sci USA 68: 1639-43.

10. Gibson D (2011) Enzymatic assembly of overlapping DNA fragments. Methods Enzymol 498: 349-61.
