# Supplementary material for: Inference of Quantitative Models of Bacterial Promoters from Time-Series Reporter Gene Data
Source: PLoS Comput Biol. 2015 Jan 15;11(1):e1004028. doi: 10.1371/journal.pcbi.1004028 (PMC4295839; doi:10.1371/journal.pcbi.1004028)
Supplement: S2 Text — (PDF) [file pcbi.1004028.s002.pdf]

Supplementary Text S2: Measurement models for reporter gene data[a]

Diana Stefan[1,2], Corinne Pinel[1,2], Stéphane Pinhal[1,2], Eugenio Cinquemani[1], Johannes Geiselmann[1,2], Hidde de Jong[1,*]
1 INRIA Grenoble – Rhône-Alpes, Grenoble, France
2 Laboratoire Interdisciplinaire de Physique (LIPhy, CNRS UMR 5588), Université Joseph Fourier, Grenoble, France
∗ Corresponding author. INRIA Grenoble—Rhône-Alpes, 655 avenue de l'Europe, Monbonnot, 38334 Saint-Ismier Cedex, France. Tel.: +33476615335, Fax: +33456527120, Email: Hidde.de-Jong@inria.fr

The reconstruction of biologically-relevant quantities from reporter gene data requires measurement models [1]. The basic measurement model underlying the analysis of the data in this paper describes the expression of the gene of interest in two steps:

$$\frac{d}{dt}m(t) = g(t) - (\mu(t) + \gamma_m)\, m(t), \quad m(0) = m_0, \tag{1}$$

$$\frac{d}{dt}p(t) = \kappa_p\, m(t) - (\mu(t) + \gamma_p)\, p(t), \quad p(0) = p_0, \tag{2}$$

where $m(t), p(t)$ are the mRNA and protein concentrations, respectively, $\mu(t)$ is the time-varying growth rate, κ_p is the protein synthesis rate constant, and γ_m, γ_p are the degradation constants of mRNA and protein, respectively. We write a similar measurement model for the reporter protein:

$$\frac{d}{dt}n(t) = g(t) - (\mu(t) + \gamma_n)\, n(t), \quad n(0) = n_0, \tag{3}$$

$$\frac{d}{dt}r(t) = \kappa_r\, n(t) - (\mu(t) + \gamma_r)\, r(t), \quad r(0) = r_0, \tag{4}$$

with analogous meanings for the variables and parameters. Notice that by construction of the transcriptional fusions, the mRNA synthesis rates or promoter activities of the gene of interest and the reporter gene are equal. This promoter activity is denoted by $g(t)$.

Two common assumptions make it possible to simplify the above models. First of all, typical mRNA half-lives in bacteria are on the order of a few minutes [2], whereas typical cell doubling times range from tens of minutes to hours [3, 4]. This motivates $\gamma_m, \gamma_n \gg \mu(t)$. Second, the mRNA concentrations evolve on a much faster time-scale than the protein concentrations, so that the former are assumed to be in quasi-steady state: $dm(t)/dt = dn(t)/dt = 0$. As a consequence, $m(t) = g(t)/\gamma_m$ and $n(t) = g(t)/\gamma_n$, and the models of Eqs. 1-4 in this text simplify to the following reduced models:

$$\frac{d}{dt}p(t) = \hat{k}_p\, g(t) - (\mu(t) + \gamma_p)\, p(t), \quad p(0) = p_0, \tag{5}$$

$$\frac{d}{dt}r(t) = \hat{k}_r\, g(t) - (\mu(t) + \gamma_r)\, r(t), \quad r(0) = r_0, \tag{6}$$

with $\hat{k}_p = \kappa_p/\gamma_m$ and $\hat{k}_r = \kappa_r/\gamma_n$.

[a] This text contains supplementary information for the paper "Inference of quantitative models of bacterial promoters from time-series reporter gene data".

We define

$$f(t) = \hat{k}_r \, g(t). \tag{7}$$

This quantity denotes the synthesis rate of the reporter protein and is proportional to the synthesis rate of the protein of interest, with proportionality constant $\alpha = (\kappa_r/\kappa_p)(\gamma_m/\gamma_n)$, *i.e.*,

$$f(t) = \alpha \, \hat{k}_p \, g(t). \tag{8}$$

Therefore, if $\kappa_p = \kappa_r$ (true for translational fusions) and $\gamma_m = \gamma_n$, then $f(t)$ also equals the synthesis rate of the protein of interest. Notice that this quantity is the starting-point for the derivation of Eq. 1 in the main text, the regulation function of FliA-dependent genes (Text S4).

As explained in Text S3, $f(t)$ can be directly computed from the absorbance and fluorescence signals. The quantity is usually called promoter activity in the literature, motivated by the fact that it is proportional to $g(t)$. Interestingly, this quantity is also proportional to the mRNA concentration of the gene of interest. This simply follows from the fact that $f(t)$ is proportional to $\hat{k}_p \, g(t)$ and the latter expression equals $k_p \, m(t)$ by Eq. 2 in this text. In the main text we refer to $f(t)$ as the promoter activity, or more generally, the activity of the gene.

One of the limitations of the above measurement model is that it assumes that k_p, k_r are constants and do not depend on the time-varying activity of the ribosomes. The model also does not distinguish between the contributions of specific transcription regulators and the activity of RNA polymerase to the promoter activity $g(t)$. In order to address these limitations, we can easily generalize the measurement models by positing

$$g(t) = k_m \, g_{global}(t) \, g_{specific}(t), \tag{9}$$

and by replacing k_p by $k_p(t)$, and k_r by $k_r(t)$. Analogously to Eq. 7 in this text, we define a generalized expression for the synthesis rate of the reporter protein:

$$f(t) = \left(k_m \, \hat{k}_r(t) \, g_{global}(t) \right) \, g_{specific}(t), \tag{10}$$

which is decomposed in a part due to the activity of the gene expression machinery $(k_m \, \hat{k}_r(t) \, g_{global}(t))$ and a part due to specific effects of transcription regulators $(g_{specific}(t))$. By the same reasoning as before, this expression is proportional to the synthesis rate of the protein of interest (with proportionality constant $(\kappa_r/\kappa_p)(\gamma_m/\gamma_n)$).

Now consider a reporter gene with a constitutive promoter that has the same ribosome-binding site as the reporter of the gene of interest. In this case, following Eq. 9 in this text, we have

$$g_{const}(t) = k_m^{const} \, g_{global}(t), \tag{11}$$

and, correspondingly, analogously to Eq. 10 in this text,

$$f_{const}(t) = k_m^{const} \, \hat{k}_r(t) \, g_{global}(t), \tag{12}$$

We therefore find

$$f(t) = \left(\frac{k_m}{k_m^{const}} \, f_{const}(t) \right) \, g_{specific}(t). \tag{13}$$

That is, when measuring both $f(t)$ (by means of the reporter of the gene of interest) and $f_{const}(t)$ (by means of the reporter of a constitutively expressed gene), we can separate global physiological effects due to the activity of the gene expression machinery and specific effects due to transcription factors and other regulators. This idea underlies the gene regulation function of Eq. 3 in the main text, as explained in Text S4.

References

1. de Jong H, Ranquet C, Ropers D, Pinel C, Geiselmann J (2010) Experimental and computational validation of models of fluorescent and luminescent reporter genes in bacteria. BMC Syst Biol 4: 55.

2. Bernstein J, Khodursky A, Lin PH, Lin-Chao S, Cohen S (2002) Global analysis of mRNA decay and abundance in *Escherichia coli* at single-gene resolution using two-color fluorescent DNA microarrays. Proc Natl Acad Sci USA 99: 9697-702.

3. Larrabee K, Phillips J, Williams G, Larrabee A (1980) The relative rates of protein synthesis and degradation in a growing culture of *Escherichia coli*. J Biol Chem 255: 4125-30.

4. Mosteller R, Goldstein R, Nishimoto K (1980) Metabolism of individual proteins in exponentially growing *Escherichia coli*. J Biol Chem 255: 2524-32.
