# Supplementary material for: Inference of Quantitative Models of Bacterial Promoters from Time-Series Reporter Gene Data
Source: PLoS Comput Biol. 2015 Jan 15;11(1):e1004028. doi: 10.1371/journal.pcbi.1004028 (PMC4295839; doi:10.1371/journal.pcbi.1004028)
Supplement: S3 Text — (PDF) [file pcbi.1004028.s003.pdf]

Supplementary Text S3: Data analysis[a]

Diana Stefan[1,2], Corinne Pinel[1,2], Stéphane Pinhal[1,2], Eugenio Cinquemani[1], Johannes Geiselmann[1,2], Hidde de Jong[1,*]
1 INRIA Grenoble – Rhône-Alpes, Grenoble, France
2 Laboratoire Interdisciplinaire de Physique (LIPhy, CNRS UMR 5588), Université Joseph Fourier, Grenoble, France
∗ Corresponding author. INRIA Grenoble—Rhône-Alpes, 655 avenue de l'Europe, Monbonnot, 38334 Saint-Ismier Cedex, France. Tel.: +33476615335, Fax: +33456527120, Email: Hidde.de-Jong@inria.fr

As described in the *Methods and materials* section of the main text, *in-vivo* and real-time gene expression profiles were obtained by means of fluorescent reporter gene systems monitored in an automated, thermostated microplate reader. In a typical experiment, we followed 96 cultures in parallel, over 16 h. The absorbance measured at 600 nm quantifies the biomass, while the fluorescence signal emitted at 520 nm, when excited at 485 nm, is proportional to the number of GFP molecules. In this section we describe how, by means of the measurement models of Text S2, we derive promoter activities and protein concentrations from the absorbance and fluorescence data. The procedures used in this study are those used in [1], with some small modifications.

Background subtraction

We first corrected the absorbance for background absorbance of the growth medium. The corrected absorbance signal $A(t)$ is computed as

$$A(t) = A_u(t) - A_b(t), \tag{1}$$

where $A_u(t)$ is the primary absorbance signal and $A_b(t)$ the absorbance of the growth medium (M9 or LB, depending on the experiment). The fluorescence signal was corrected for autofluorescence generated by wild-type bacteria carrying the (non-functional) pUA66*gfp* plasmid or no plasmid at all (in practice these two measures of the autofluorescence gave the same result). The autofluorescence depends on the (time-varying) population size. Since the culture generating the fluorescence signal of interest and the culture generating the autofluorescence signal may not be exactly synchronized, direct subtraction of the autofluorescence background is not always possible. We used a calibration procedure, such that the corrected signal $I(t)$ is defined by

$$I(t) = I_u(t) - s(A(t)), \tag{2}$$

where $I_u(t)$ is the primary fluorescence level and s a calibration function, mapping absorbance levels to autofluorescence levels. The calibration function is obtained by fitting a cubic smoothing spline to the autofluorescence generated by bacteria carrying the (non-functional) pUA66*gfp* plasmid or no plasmid at all as a function of the absorbance. Splines have the advantage that they can be evaluated for any absorbance within the observed range and easily extrapolated beyond this range. Figure 1 in this text gives an example of background correction of absorbance and fluorescence data, in the case of the reporter of *tar* in the $\Delta cpxR$ mutant strain.

[a]This text contains supplementary information for the paper "Inference of quantitative models of bacterial promoters from time-series reporter gene data".

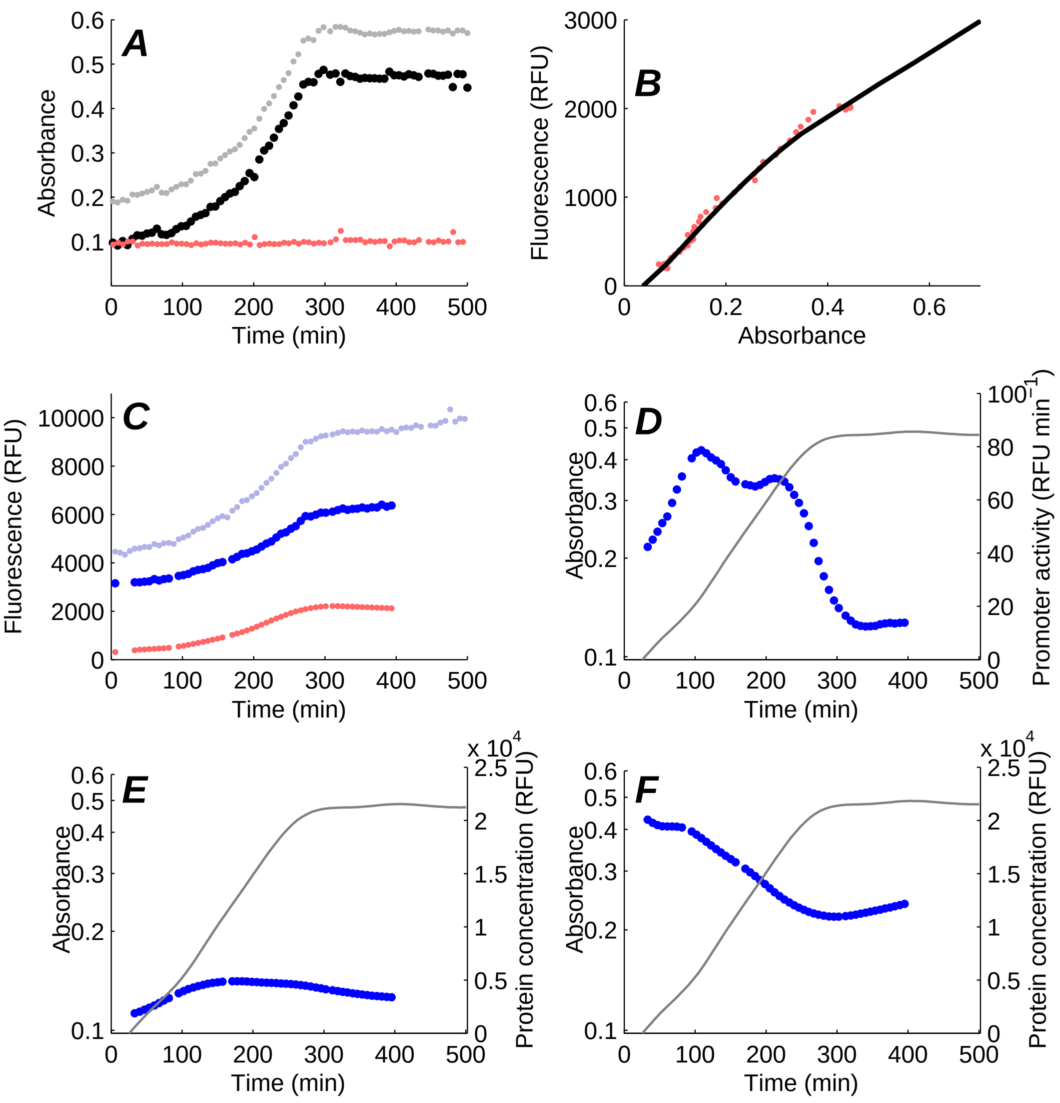

Figure 1. Illustration of data analysis procedures. Absorbance and fluorescence data acquired with the $\Delta cpxR$ mutant strain carying a pUA66tar-gfp plasmid, grown in M9 with glucose. *A:* Primary (uncorrected) absorbance (•, grey), background absorbance (•, red), and corrected absorbance (•, black). *B:* Calibration curve obtained by measuring the autofluorescence of the wild-type strain without plasmid. Primary fluorescence data are plotted against (corrected) absorbance data and the curve is obtained by fitting a smoothing spline. *C:* Primary fluorescence data (•, grey), and the corrected fluorescence (•, blue) obtained after subtracting the fluorescence of the background (•, red) as in Eq. 2 in this text. *D:* Promoter activity of tar (•, blue) computed from the corrected absorbance (-, grey) and corrected fluorescence by means of Eq. 6 in the main text. *E:* Concentration of Tar (•, blue) computed for a half-life of 2 h from the corrected absorbance (-, grey) using Eq. 7 in the main text. *F:* Concentration of Tar (•, blue) computed for a half-life of 18 h from the corrected absorbance (-, grey) using Eq. 7 in the main text.

Computation of promoter activity and protein concentration

The corrected absorbance and fluorescence data were used to compute promoter activities (synthesis rates) and protein concentrations, following the measurement models in Text S2. From Eqs. 6-7 in Text S2 it follows that

$$f(t) = \frac{d}{dt} r(t) + (\mu(t) + \gamma_r)\, r(t). \tag{3}$$

The growth rate $\mu(t)$ can be estimated from the absorbance, that is,

$$\mu(t) = \frac{d}{dt} A(t) \frac{1}{A(t)} = \frac{d \ln A(t)}{dt}. \tag{4}$$

The time-varying GFP concentration in the bacterial population, $r(t)$, can also be estimated from the absorbance and fluorescence, making the usual assumptions that the fluoresence is proportional to the number of GFP molecules and the absorbance proportional to the biomass:

$$r(t) \sim \frac{I(t)}{A(t)}. \tag{5}$$

We arbitrarily set the proportionality constant in Eq. 5 in this text to 1, thus expressing the reporter protein concentration in units RFU (and the synthesis rate in units RFU min^{-1}). Substituting the expressions for $r(t)$ and $\mu(t)$ into Eq. 3 in this text yields [2]:

$$\begin{aligned} f(t) &= \frac{dI(t)}{dt} \frac{1}{A(t)} - \frac{dA(t)}{dt} \frac{I(t)}{A(t)^2} + \left(\frac{dA(t)}{dt} \frac{1}{A(t)} + \gamma_r \right) \frac{I(t)}{A(t)} \\ &= \frac{\frac{d}{dt} I(t)}{A(t)} + \gamma_r \frac{I(t)}{A(t)}. \end{aligned}$$

This is Eq. 6 in the main text. The definition is equivalent to other definitions in the literature [3] when $\mu(t) >> \gamma_r$. The expression is evaluated using estimates of $A(t)$, $I(t)$, and $dI(t)/dt$ obtained by means of cubic smoothing splines, as described in the main text. Figure 1D in this text shows the promoter activity of *tar* computed from the data in panels A and C.

In order to reconstruct the concentration of a protein of interest, we again use the models of Text S2, in particular Eq. 5. The term $\hat{k}_p\, g(t)$ was seen to be proportional by a factor α to $f(t)$, following Eq. 8 in Text S2. We arbitrarily set the proportionality constant in Eq. 8 in Text S2 to 1, giving rise to Eq. 7 of the main text:

$$\frac{d}{dt} p(t) = f(t) - (\gamma_p + \mu(t))\, p(t), \quad p(0) = p_0.$$

With the definition of the initial protein concentration as $p^0 = f(T)/\gamma_p$, as explained in the same section of the main text, setting α to 1 has the effect of fixing a scaling factor for $p(t)$. Since the units of $p(t)$ are relative, the scaling factor has no consequences for the interpretation of the results. $p(t)$ was computed by numerically solving the integral, using the smoothing-spline estimates of $A(t)$, $I(t)$, $dA(t)/dt$, and $dI(t)/dt$. Figure 1E-F in this text shows the concentration of Tar in a $\Delta cpxR$ deletion strain, for two different half-lives of the protein (and thus degradation constants γ_p).

References

1. Berthoumieux S, de Jong H, Baptist G, Pinel C, Ranquet C, et al. (2013) Shared control of gene expression in bacteria by transcription factors and global physiology of the cell. Mol Syst Biol 9: 634.

2. de Jong H, Ranquet C, Ropers D, Pinel C, Geiselmann J (2010) Experimental and computational validation of models of fluorescent and luminescent reporter genes in bacteria. BMC Syst Biol 4: 55.

3. Ronen M, Rosenberg R, Shraiman B, Alon U (2002) Assigning numbers to the arrows: Parameterizing a gene regulation network by using accurate expression kinetics. Proc Natl Acad Sci USA 99: 10555-60.
