# Supplementary material for: Inference of Quantitative Models of Bacterial Promoters from Time-Series Reporter Gene Data
Source: PLoS Comput Biol. 2015 Jan 15;11(1):e1004028. doi: 10.1371/journal.pcbi.1004028 (PMC4295839; doi:10.1371/journal.pcbi.1004028)
Supplement: S4 Text — (PDF) [file pcbi.1004028.s004.pdf]

Supplementary Text S4: Derivation of regulation function of motility genes[a]

Diana Stefan[1,2], Corinne Pinel[1,2], Stéphane Pinhal[1,2], Eugenio Cinquemani[1], Johannes Geiselmann[1,2], Hidde de Jong[1,*]
1 INRIA Grenoble – Rhône-Alpes, Grenoble, France
2 Laboratoire Interdisciplinaire de Physique (LIPhy, CNRS UMR 5588), Université Joseph Fourier, Grenoble, France
∗ Corresponding author. INRIA Grenoble—Rhône-Alpes, 655 avenue de l'Europe, Monbonnot, 38334 Saint-Ismier Cedex, France. Tel.: +33476615335, Fax: +33456527120, Email: Hidde.de-Jong@inria.fr

We develop a kinetic model for the regulation of the expression of *tar* as a function of the total concentrations of FliA and FlgM (see Figure 1 in main text). The model is based on a quasi-equilibrium approximation of the mass-action kinetics for the formation of the FliA·FlgM complex, and a phenomenological Hill-type regulatory law of *tar* expression by free FliA.

Let $p_{A,free}$, $p_{M,free}$ and p_{AM} denote the concentrations of free FliA, free FlgM and FliA·FlgM, in that order, and let p_A, p_M denote total concentrations of FliA and FlgM. Assuming complex formation and dissociation are fast events relative to gene expression and protein degradation, we make the approximation

$$\frac{d}{dt} p_{AM} = k^+ p_{A,free} \cdot p_{M,free} - k^- p_{AM} = 0,$$

with $k^- > 0$ and $k^+ > 0$. Using the facts that $p_A = p_{A,free} + p_{AM}$ and $p_M = p_{M,free} + p_{AM}$, substitution into the above to eliminate $p_{M,free}$ and p_{AM} from the equation yields

$$k^+ p_{A,free} \cdot \big(p_M - (p_A - p_{A,free})\big) - k^-(p_A - p_{A,free}) = 0,$$

which is a second-order polynomial equation in $p_{A,free}$. The solution of the equation that satisfies $0 \leq p_{A,free} \leq p_A$ is

$$p_{A,free}(p_A, p_M) = \frac{1}{2}\left(\sqrt{(K + p_M - p_A)^2 + 4Kp_A} - (K + p_M - p_A)\right),$$

with $K = k^-/k^+$, which is a function of the concentrations p_A and p_M. In accordance with Figure 1 of the main text, only the free FliA molecules regulate the expression of p*tar*, and we quantify the regulatory effect by the law

$$\frac{p^n_{A,free}}{p^n_{A,free} + \theta^n},$$

with $n \geq 1$. Multiplying by maximal synthesis rate k_1 and adding basal (unregulated) synthesis rate k_0 leads to model Eq. 1- 2 in the main text.

Note that, in accordance with the expected regulatory pattern, the function

$$k_0 + k_1 \cdot \frac{p^n_{A,free}(p_A, p_M)}{p^n_{A,free}(p_A, p_M) + \theta^n}$$

[a]This text contains supplementary information for the paper "Inference of quantitative models of bacterial promoters from time-series reporter gene data".

is increasing in p_A and decreasing in p_M. To verify this, it suffices to show that derivatives with respect to p_A and p_M are nonnegative and nonpositive, respectively. Ignoring k_0 and k_1 without loss of generality, the derivative of $p_{A,free}$ with respect to p_A can be written as

$$\frac{1}{2} + \frac{1}{2} \cdot \frac{-(p_M - p_A + K) + 2K}{\sqrt{(p_M - p_A + K)^2 + 4Kp_A}}.$$

This expression is obviously positive if $p_M - p_A + K \leq 0$. If instead $p_M - p_A + K > 0$, note that the expression is still positive if the square of the fraction,

$$\frac{\big((p_M - p_A + K) - 2K\big)^2}{(p_M - p_A + K)^2 + 4Kp_A},$$

is smaller than 1. But this is apparent since, under $p_M - p_A + K > 0$, the numerator is no bigger than $(p_M - p_A + K)^2$, whereas the denominator is no smaller than the same quantity. Similarly, the derivative of $p_{A,free}$ with respect to p_M can be written as

$$-\frac{1}{2} + \frac{1}{2} \cdot \frac{p_M - p_A + K}{\sqrt{(p_M - p_A + K)^2 + 4Kp_A}}.$$

This expression is obviously negative if $(p_M - p_A + K) \leq 0$. If instead $(p_M - p_A + K) > 0$, note that the square root is no smaller than $(p_M - p_A + K)$, hence the rightmost fraction is no bigger than 1, *i.e.* the overall expression is again negative.

The additional regulatory effect of global physiological effects is quantified via further multiplication by a function $f_{const}(t)$, as in the *Results* section of the main text. Monotonicity with respect to p_A and p_M remains unchanged. In addition, f is increasing in f_{const}. In all cases, the model depends on the (nonnegative) parameters k_0, k_1, n, θ, K.
