# Supplementary material for: Inference of Quantitative Models of Bacterial Promoters from Time-Series Reporter Gene Data
Source: PLoS Comput Biol. 2015 Jan 15;11(1):e1004028. doi: 10.1371/journal.pcbi.1004028 (PMC4295839; doi:10.1371/journal.pcbi.1004028)
Supplement: S5 Text — (PDF) [file pcbi.1004028.s005.pdf]

Supplementary Text S5: Computation of minimal consistent sign patterns[a]

Diana Stefan[1,2], Corinne Pinel[1,2], Stéphane Pinhal[1,2], Eugenio Cinquemani[1], Johannes Geiselmann[1,2], Hidde de Jong[1,*]
1 INRIA Grenoble – Rhône-Alpes, Grenoble, France
2 Laboratoire Interdisciplinaire de Physique (LIPhy, CNRS UMR 5588), Université Joseph Fourier, Grenoble, France
∗ Corresponding author. INRIA Grenoble—Rhône-Alpes, 655 avenue de l'Europe, Monbonnot, 38334 Saint-Ismier Cedex, France. Tel.: +33476615335, Fax: +33456527120, Email: Hidde.de-Jong@inria.fr

In this section we adopt a notation analogous to the *Methods and Materials* section in the main text, where vector $x = (x_1, \dots, x_n)$ indicates concentrations of regulators (p_A, p_M), and may comprise in addition global physiological effects, depending on the context of use. What follows applies identically for all target genes of interest, *i.e. tar* in the main text. Let $f(x)$ be the promoter activity of one gene of interest. For network inference, where f is *a-priori* unknown, we use the minimal sign pattern method of [1]. The method relies on two assumptions:

1. $f(x)$ is monotone in every x_j, with $j = 1, \dots, n$.
2. A set of measurements $D = \{(\bar{x}^k, \bar{f}^k) : \ k = 1, \dots, m\}$ of the concentration vectors x and the corresponding target promoter activities f are available, along with confidence intervals $\bar{f}^k \pm \epsilon^k$ and $\bar{x}^k \pm e^k$.

Assumption 1 reflects the hypothesis that no regulator can act as an activator as well as a repressor of a given gene (see [1] and references therein), though the same regulator can be an activator for one gene and a repressor for another. We may thus define the sign pattern $\pi = (\pi_1, \dots \pi_n)$ of f by posing $\pi_j = 1$ if f is increasing in x_j, $\pi_j = -1$ if f is decreasing in x_j, and $\pi_j = 0$ if f is independent of x_j, $j = 1, \dots, n$. The sign pattern encodes the directed, signed graph of the regulation of the gene under consideration by all possible regulators in the network (compare Fig. 1*B-E* in this text). For assumption 2, data may come from several reporter gene experiments (different strains and media) and is provided in the required form by the processing of Text S3, where $(\bar{x}^k, \bar{f}^k)$ is the measurement average at time t_k, while e^k and ϵ^k are fixed to twice the standard error of the mean $(\bar{x}^k, \bar{f}^k)$.

The rationale of the procedure for eliminating hypotheses from the set of all candidate sign patterns is the following [1]. Given any two concentration vectors x' and x'', for all $j = 1, \dots, n$ the implication

$$\pi_j(x''_j - x'_j) \geq 0 \Rightarrow f(x'') \geq f(x')$$

is satisfied by the very definition of the sign pattern π of f. Therefore, for a hypothetical sign pattern $\bar{\pi}$ and perfect measurements ($\epsilon^k = e^k = 0$ for all k), any two data points $(\bar{x}', \bar{f}')$ and $(\bar{x}'', \bar{f}'')$ that falsify the implication allow one to conclude that $\bar{\pi}$ is not the sign pattern of f. In particular, if $\bar{f}'' < \bar{f}'$, the sign pattern $\bar{\pi}$ defined by $\bar{\pi}_j = 1$ if $\bar{x}''_j > \bar{x}'_j$, $\bar{\pi}_j = -1$ if $\bar{x}''_j < \bar{x}'_j$, and $\bar{\pi}_j = 0$ otherwise, is inconsistent with the data. In addition, any subpattern of $\bar{\pi}$, *i.e.* a pattern $\tilde{\pi}$ whose nonzero entries are equal to the corresponding entries of $\bar{\pi}$ (denoted with $\tilde{\pi} \sqsubseteq \bar{\pi}$), is also inconsistent with the data, since the implication above is still violated. This test is easily robustified to account for measurement uncertainties. For any data point $(\bar{x}, \bar{f}) \in D$, let $(\hat{x}, \hat{f})$ and $(\check{x}, \check{f})$ indicate the confidence bounds $\hat{f} = \bar{f} + \epsilon$ and $\check{f} = \bar{f} - \epsilon$, in the same order, and similarly $\hat{x}_j = \bar{x}_j + e_j$ and $\check{x}_j = \bar{x}_j - e_j$, with $j = 1, \dots, n$. Let the complexity C

[a]This text contains supplementary information for the paper "Inference of quantitative models of bacterial promoters from time-series reporter gene data".

of a sign pattern π be the number of nonzero entries of π. The algorithm is divided into two phases, conceptually organized as follows (see Figure 1 in this text for reference).

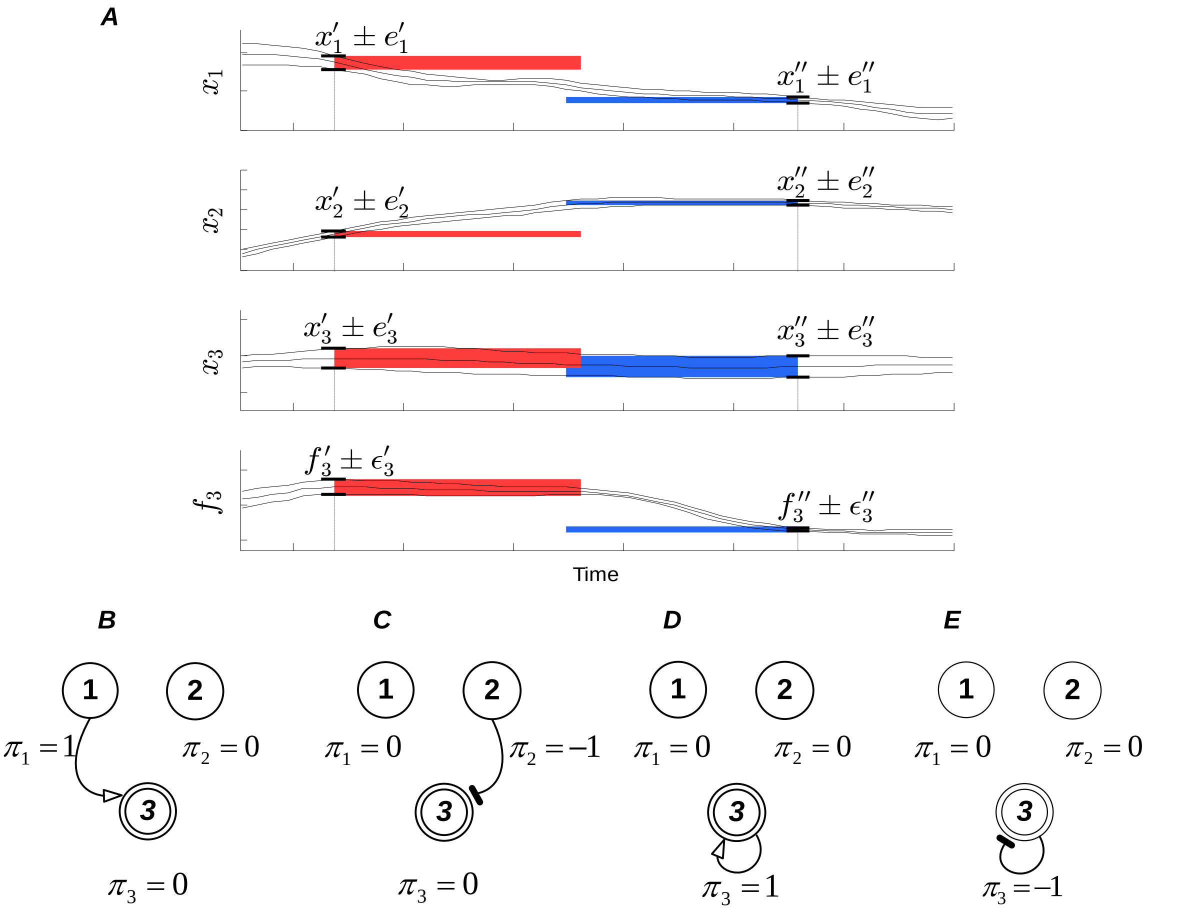

Figure 1. Computation of inconsistent and minimal consistent sign patterns from data. An example of the method explained in this text is shown for the regulation of gene 3 in a hypothetical network with genes 1,2,3. *A:* From top to bottom, example time profiles and corresponding confidence intervals (thin black lines) for the concentrations of the proteins encoded by genes 1,2,3 and the synthesis rate of gene 3. Only the two data points (x', f'_3) and (x', f''_3) are considered in this example. Non-overlapping confidence intervals of f'_3 and f''_3 (reported next to each other for ease of comparison by the red and pink shaded regions) imply $\hat{f}''_3 < \check{f}'_3$. Similarly, non-overlapping confidence intervals for x'_1, x''_1 and x'_2, x''_2 imply $\hat{x}''_1 < \check{x}'_1$ $(\bar{\pi}_1 = -1)$ and $\check{x}''_2 > \hat{x}'_2$ $(\bar{\pi}_2 = 1)$, respectively, while confidence intervals for x'_3 and x''_3 overlap $(\bar{\pi}_3 = 0)$. Whence, $\bar{\pi} = (-1, 1, 0)$. If this was the sign pattern of f_3, then $f_3(x)$ should increase for x'_1 decreasing to x''_1 and x'_2 increasing to x''_2 (x_3 is irrelevant in the hypothesis $\bar{\pi}_3 = 0$), whereas the observation says that $\hat{f}''_3 < \check{f}'_3$. Pattern $\bar{\pi} = (-1, 1, 0)$ is thus inconsistent with the data. *B - E:* Regulation patterns for gene 3, corresponding to the consistent sign patterns of f_3 deduced from the inconsistent patterns $\bar{\Pi} = \{\bar{\pi}\}$ obtained in *A*. Circles represent genes; directed arcs represent regulation of the target gene 3 by regulator j, with $j = 1, 2, 3$; arrow ends represent activation $(\pi_j = 1)$, line ends represent inhibition $(\pi_j = -1)$. Black arrows represent the minimal consistent sign patterns $(1, 0, 0)$ in *B*, $(0, -1, 0)$ in *C*, $(0, 0, 1)$ in *D*, and $(0, 0, -1)$ in *E*. Every consistent pattern is obtained from one of the cases *B - E* by turning the corresponding $\pi_j = 0$ into either $\pi_j = -1$ or $\pi_j = 1$.

Computation of inconsistent patterns $\bar{\Pi}$ from data D

- Set $\bar{\Pi} = \emptyset$
- For all pairwise different data points $(\bar{x}', \bar{f}')$ and $(\bar{x}'', \bar{f}'')$ in D:

  If $\hat{f}'' < \check{f}'$

  - Define $\bar{\pi} = (\bar{\pi}_1, \ldots, \bar{\pi}_n)$ by $\bar{\pi}_j = \begin{cases} 1, & \check{x}'' > \hat{x}' \\ -1, & \hat{x}'' < \check{x}' \\ 0, & \text{otherwise} \end{cases}$, with $j = 1, \ldots, n$
  - Include $\bar{\pi}$ in $\bar{\Pi}$
- Return $\bar{\Pi}$

At this stage, a generic pattern π is inconsistent if and only if $\pi \sqsubseteq \bar{\pi}$ for some $\bar{\pi} \in \bar{\Pi}$ [1].

Computation of minimal consistent patterns Π^* from $\bar{\Pi}$

- Set $\Pi^* = \emptyset$
- For $C = 0, 1, \ldots, n$:
  - Enumerate all possible patterns π of complexity C
  - For every such π:

    If $\nexists\ \bar{\pi} \in \bar{\Pi}$ such that $\pi \sqsubseteq \bar{\pi}$ (π is consistent), and

    If $\nexists\ \pi^* \in \Pi^*$ such that $\pi^* \sqsubseteq \pi$ (π is minimal consistent), then

    Include π in Π^*
- Return P^*

At this stage, a generic pattern π is consistent if and only if $\pi^* \sqsubseteq \pi$ for some $\pi^* \in \Pi^*$ [1].

In practice, the above operations can be made computationally efficient. Notably, in our implementation, "for" loops and "if" tests are replaced by suitable algebraic and Boolean matrix operations in Matlab. Notice also that the above method applies in the same way when the protein concentrations are replaced by promoter activities.

Intersection of analyses from different datasets

As a final comment, we discuss the situation where one has several datasets for the analysis of the same model. A natural question arises as to how joint analysis of all datasets compares with the separate analysis of the individual datasets (see for instance Figure 11 in the main text). Consider, without loss of generality, two datasets D_1 and D_2 (each being a set of couples of the type $(\bar{x}, \bar{f})$). Let Π_1^*, Π_2^* and Π^* be the set of minimal sign patterns consistent with D_1, D_2 and $D_1 \cup D_2$, in the same order. Similarly let Π_1, Π_2 and Π be the set of all patterns consistent with D_1, D_2 and $D_1 \cup D_2$, again in the same order. One can easily prove that

$$\Pi^* \subseteq \Pi \subseteq \Pi_1 \cap \Pi_2.$$

The relation between Π_1^*, Π_2^* and Π^* is less obvious. Consider for example a regulation function with three putative regulators, and suppose that, upon analysis of D_1 and D_2, one finds $\Pi_1^* = \{(1, 0, 0)\}$ and $\Pi_2^* = \{(0, -1, 0)\}$. Then Π_1 is composed of all patterns with a 1 in first position, and Π_2 is composed of all

patterns with a -1 in second position. Then Π is contained in the set of all patterns $\pi = (\pi_1, \pi_2, \pi_3)$ with $\pi_1 = 1$ and $\pi_2 = -1$ and any π_3. Assuming that no patterns are invalidated, based on the comparison of one data point in D_1 and one in D_2, equality $\Pi = \Pi_1 \cap \Pi_2$ holds, whence the minimal consistent sign pattern set corresponding to Π is $\Pi^* = \{(1, -1, 0)\}$.

References

1. Porreca R, Cinquemani E, Lygeros J, Ferrari-Trecate G (2010) Identification of genetic network dynamics with unate structure. Bioinformatics 26: 1239-45.
