# Supplementary material for: Inference of Quantitative Models of Bacterial Promoters from Time-Series Reporter Gene Data
Source: PLoS Comput Biol. 2015 Jan 15;11(1):e1004028. doi: 10.1371/journal.pcbi.1004028 (PMC4295839; doi:10.1371/journal.pcbi.1004028)
Supplement: S6 Text — (PDF) [file pcbi.1004028.s006.pdf]

Supplementary Text S6: Validation of reporter gene data using qRT-PCR[a]

Diana Stefan[1,2], Corinne Pinel[1,2], Stéphane Pinhal[1,2], Eugenio Cinquemani[1], Johannes Geiselmann[1,2], Hidde de Jong[1,*]

1 INRIA Grenoble – Rhône-Alpes, Grenoble, France

2 Laboratoire Interdisciplinaire de Physique (LIPhy, CNRS UMR 5588), Université Joseph Fourier, Grenoble, France

∗ Corresponding author. INRIA Grenoble—Rhône-Alpes, 655 avenue de l'Europe, Monbonnot, 38334 Saint-Ismier Cedex, France. Tel.: +33476615335, Fax: +33456527120, Email: Hidde.de-Jong@inria.fr

According to Eq.1 of Text S2, the ratio of the promoter activities f_1, f_2 of two genes is proportional to the ratio of the mRNA concentrations m_1, m_2, that is,

$$\frac{f_1(t)}{f_2(t)} = \frac{k_{p,1}}{k_{p,2}} \frac{m_1(t)}{m_2(t)}. \tag{1}$$

Measuring gene expression by qRT-PCR allows the relative abundances of the mRNA of a target gene to be quantified with respect to the mRNA of a reference gene [1]. This provides a direct way to verify if the relative promoter activities measured with reporter genes are confirmed by another, independent experimental method. We compared the promoter activity of *tar*, as an example of a motility gene, with the activity of the constitutive pRM promoter. The validation of the ratio f_{tar}/f_{pRM} was carried out by means of the WTpRM strain, a modified BW25113 strain carrying a natural copy of *tar* and a transcriptional fusion of the pRM promoter with a *g*fp gene inserted into the *intS* locus on the chromosome (Text S1). qRT-PCR was used to quantify the relative abundances of *tar* and *gfp* mRNA, using a previously validated qPCR protocol [2].

We took 5 μL samples at 11 time-points from cultures of the WTpRM strain, growing in a microplate under the conditions described in the *Materials and methods* section of the main text. Total mRNA was protected using the RNAprotect Bacteria Reagent (Quiagen) and then extracted using the RNeasy mini kit (Quiagen) according to the protocols of the manufacturer. The RNA samples were then treated using the turbo DNAse (Ambion) to avoid DNA contamination. Approximately 1 μg of RNA for each of the 11 time-points was reverse transcribed using SuperScript II Reverse Transcriptase (Invitrogen). The cDNA synthesized samples were diluted 10x into MESA Green qPCR Master Mix (Eurogentec), supplemented with primers for the *flgM* and *gfp* genes. Quantitative PCR was performed in a StepOnePlus Real-Time PCR System (Applied Biosystems) according to the instructions of the manufacturer. Briefly, 5 μL reaction mixtures were incubated for 10 min at 95 ^{0}C and 40 PCR cycles (15 s at 95 ^{0}C, 10 s at 62 ^{0}C and 10 s at 70 ^{0}C). PCRs were run in quadruplicate. Raw data were transformed into threshold cycle (C_T) values. PCR amplification efficiencies for *tar* and *gfp* were determined by constructing standard curves from serial dilutions.

The results were analyzed by computing $m_1(t)/m_2(t)$ at the sample time-points t with respect to $m_1(t_0)/m_2(t_0)$, the same quantity at a reference time-point t_0 [1]:

$$q(t) = \frac{m_1(t)/m_1(t_0)}{m_2(t)/m_2(t_0)} = \frac{E_{gfp}^{\Delta C_T^{gfp}}}{E_{tar}^{\Delta C_T^{tar}}}, \tag{2}$$

where C_T^{gfp} and C_T^{tar} are the measured C_T values for *gfp* and *tar*, respectively, $\Delta C_T^{gfp}(t) = C_T^{gfp}(t) - C_T^{gfp}(t_0)$, $\Delta C_T^{tar}(t) = C_T^{tar}(t) - C_T^{tar}(t_0)$. As our reference time-point, we chose a measurement during

[a]This text contains supplementary information for the paper "Inference of quantitative models of bacterial promoters from time-series reporter gene data".

exponential growth on glucose. As a consequence, the changes in mRNA abundance are relative to the mRNA abundance in exponential phase. The efficiencies were measured to be nearly 109% for *gfp* ($E_{gfp} = 2.09$) and 105% for *tar* ($E_{tar} = 2.05$).

From Eq. 1 it follows that

$$q(t) = \frac{f_1(t)/f_1(t_0)}{f_2(t)/f_2(t_0)}. \quad (3)$$

The right-hand side of this equation can be computed from the measured promoter activities (Text S2). Figure 1 in this text compares the value of $q(t)$ measured by means of reporter genes and qRT-PCR. As can be seen, there is a very good qualitative and quantitative correspondence between the two independent measurements of q. In other words, the qRT-PCR measurements support the reporter gene measurements.

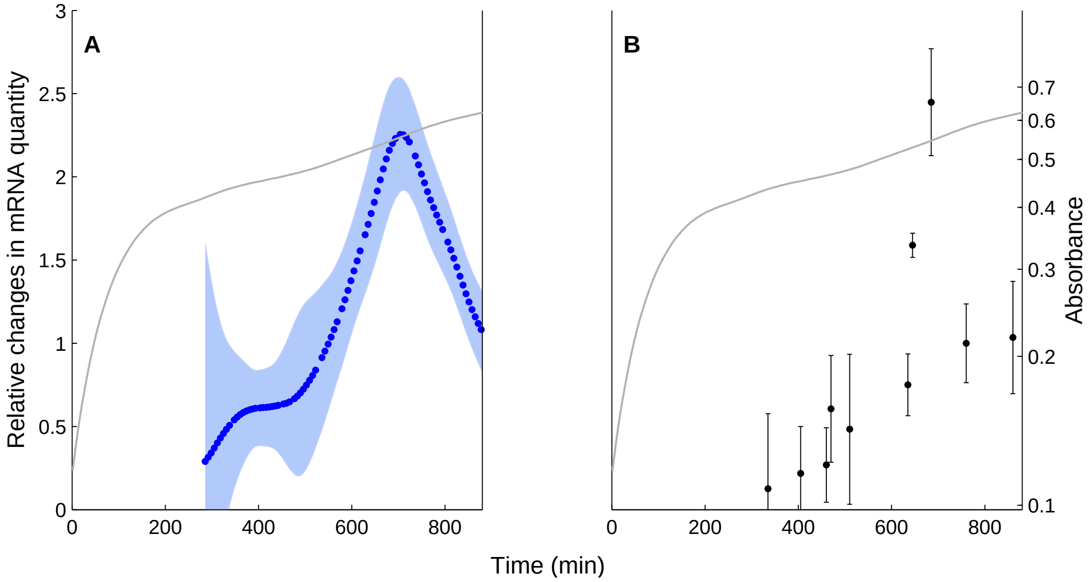

Figure 1. Validation of the reporter gene measurements using quantitative RT-PCR. *A*: Promoter activity of *tar* with respect to the activity of the constitutive phage promoter computed by means of Eq. 2 in this text (solid, blue). The promoter activities were derived from corrected absorbance and fluorescence, measured using plasmids expressing the GFP reporter under the control of the pRM and the *tar* promoters, respectively. The shaded regions represent confidence intervals computed as ± twice the standard error of the mean of 5 replicates. *B*: Abundance of *tar* mRNA with respect to the abundance of *gfp* mRNA transcribed from the pRM promoter (•, black). The mRNA quantities were measured using qRT-PCR and the ratio computed from Eq. 3 in this text. The results obtained by means of two independent techniques are in good agreement. The error bars were computed from the standard error of the mean of 4 replicates. The absorbances have been also plotted on the figures (solid line, grey).

References

1. Pfaffl M (2001) A new mathematical model for relative quantification in real-time RT-PCR. Nucleic Acids Res 29: e45.

2. Lee C, Kim J, Shin S, Hwang S (2004) Absolute and relative QPCR quantification of plasmid copy number in *Escherichia coli*. J Biotechnol 123: 273-80.
