# Supplementary material for: Inference of Quantitative Models of Bacterial Promoters from Time-Series Reporter Gene Data
Source: PLoS Comput Biol. 2015 Jan 15;11(1):e1004028. doi: 10.1371/journal.pcbi.1004028 (PMC4295839; doi:10.1371/journal.pcbi.1004028)
Supplement: S7 Text — (PDF) [file pcbi.1004028.s007.pdf]

Supplementary Text S7: Additional data, analysis results and fits[a]

Diana Stefan[1,2], Corinne Pinel[1,2], Stéphane Pinhal[1,2], Eugenio Cinquemani[1], Johannes Geiselmann[1,2], Hidde de Jong[1,*]

1 INRIA Grenoble – Rhône-Alpes, Grenoble, France

2 Laboratoire Interdisciplinaire de Physique (LIPhy, CNRS UMR 5588), Université Joseph Fourier, Grenoble, France

∗ Corresponding author. INRIA Grenoble—Rhône-Alpes, 655 avenue de l'Europe, Monbonnot, 38334 Saint-Ismier Cedex, France. Tel.: +33476615335, Fax: +33456527120, Email: Hidde.de-Jong@inria.fr

We present below additional data from reporter gene experiments, fits of regulation function to reporter gene data, and results from the computations of minimal sign patterns.

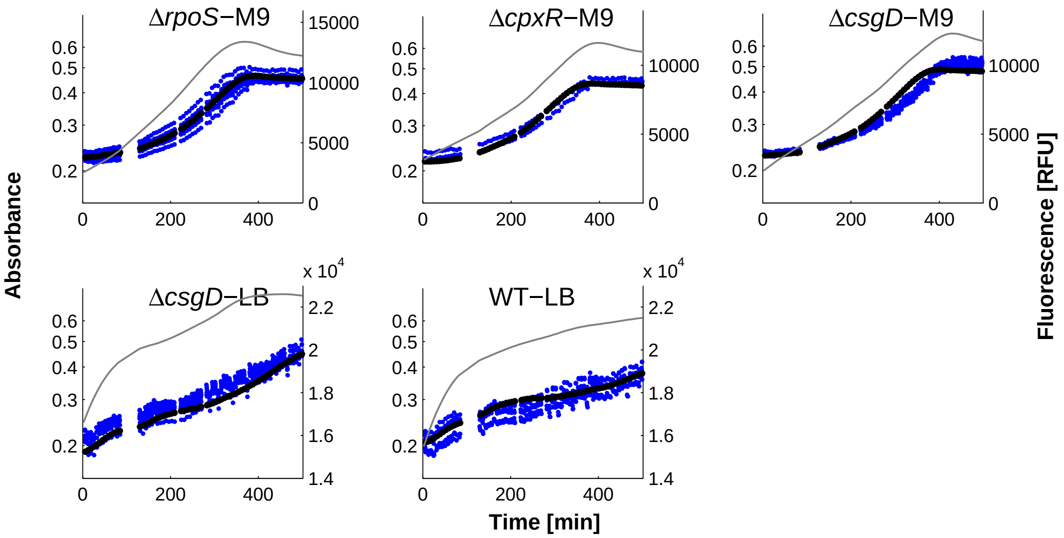

Figure 1. Monitoring the expression of *flgA* promoter. The figure shows the fluorescence profiles corresponding to the activity of *flgA* (•, blue) and the background fluorescence (•, black). The expression of this class 2 promoter has been observed in all strains and growth media considered in this study ($\Delta rpoS$, $\Delta cpxR$, $\Delta csgD$-M9, $\Delta csgD$-LB and WT-LB). Since the fluorescence signal is not distinguishable from the background fluorescence in any of the conditions, gene expression from *flgA* promoter can be ignored. Absorbance profiles (solid line, grey) show that growth conditions are similar to those in the experiments monitoring the expression from *flgM* promoter.

[a]This text contains supplementary information for the paper "Inference of quantitative models of bacterial promoters from time-series reporter gene data".

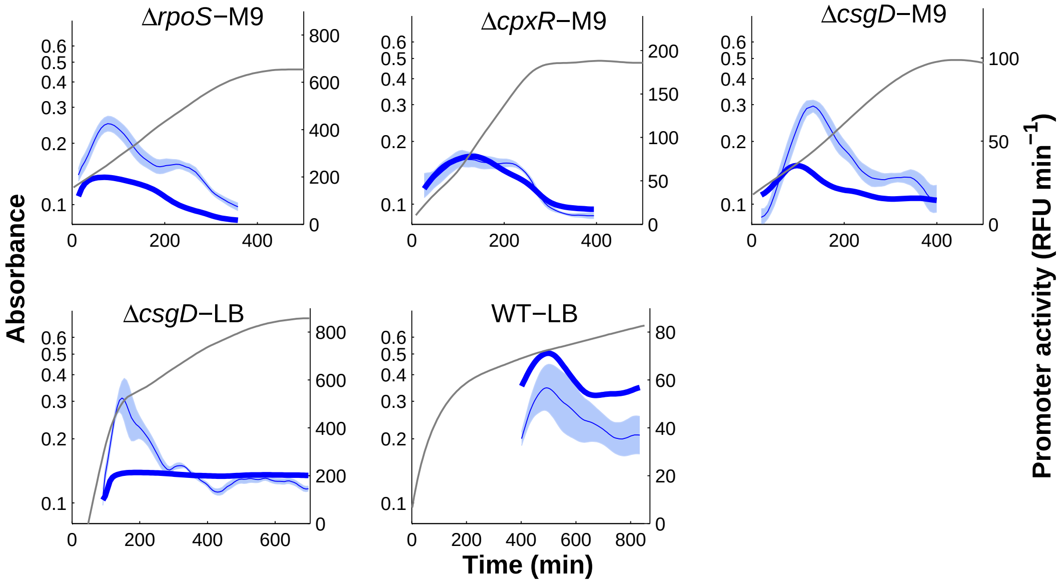

Figure 2. Fits of regulation function of tar to reporter gene data when reconstructing protein concentrations from the reporter gene data and ignoring global physiological effects. The regulation function of Eqs. 1-2 of the main text was fit to the data using the promoter activity for *tar* (Figure 3 of the main text) and concentrations of FliA and FlgM reconstructed from the activities of their promoters for physiologically realistic half-lives (Figure 8 of the main text and Figure 5 in this text). Model predictions are in dark blue (thick solid line), *tar* reporter data are in light blue (thin solid line and shaded area). The parameters were estimated using a multistart global optimization algorithm (see *Methods and materials* in main text for details). The best fit is shown, for measured half-lives of FliA and FlgM of 30 min and 18 min, respectively (solid line, $Q = 36.3$, $(k_0, k_1, n, \theta, K) = (9.4, 252, 1.4, 5675, 447499)$).

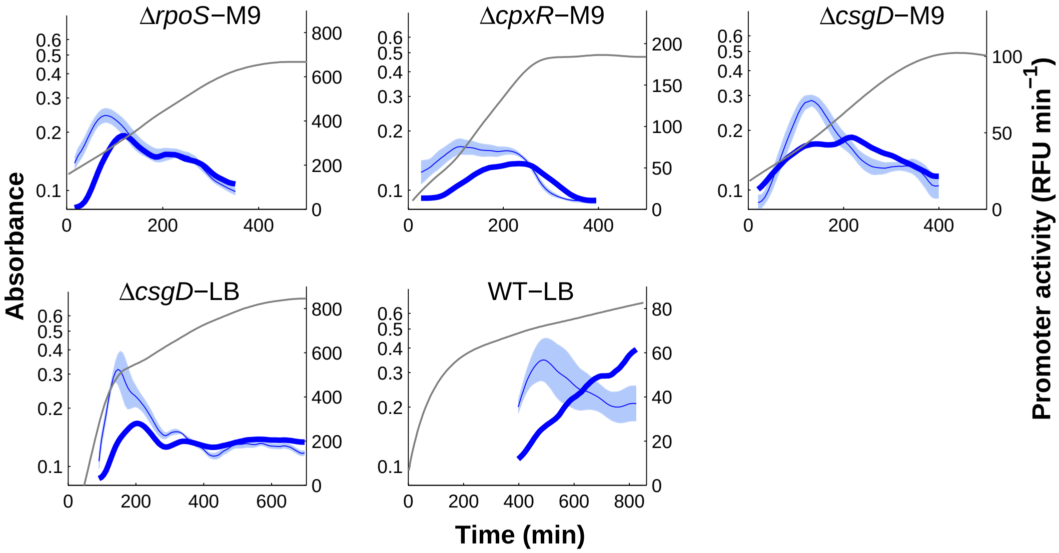

Figure 3. Fits of regulation function of tar to reporter gene data when reconstructing protein concentrations from the reporter gene data for stable half-lives and including global physiological effect. As in Figure 9 of the main text the regulation function of Eqs. 2-3 of the main text was used but now the half-lives are equal to the half-life of the reporter. Model predictions are in dark blue (thick solid line), *tar* reporter data are in light blue (thin solid line and shaded area). The parameters were estimated using a multistart global optimization algorithm (see *Methods and materials* in main text for details). The best fit is shown, for very stable half-lives of FliA and FlgM of 18 h (solid line, $Q = 31.4$, $(k_0, k_1, n, \theta, K) = (0.28, 4.8, 2.3, 12941, 80872)$).

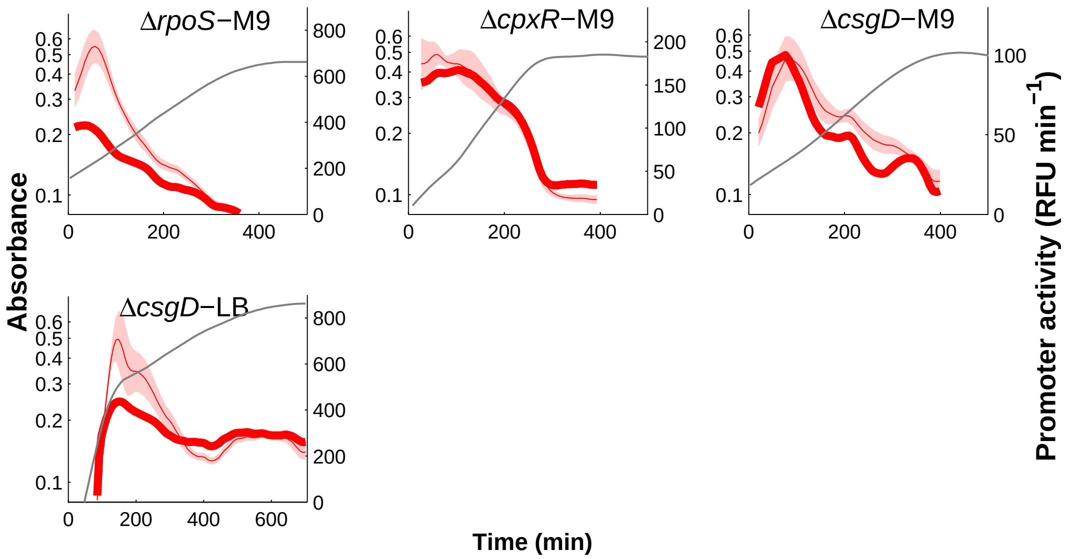

Figure 4. Fits of regulation function of $flgM$ to reporter gene data when replacing protein concentrations by promoter activities. The regulation function of Eqs. 1-2 of the main text was fit using the promoter activities for *fliA*, and *flgM* shown in Figure 3 of the main text, where the latter two replace the concentrations of FliA and FlgM, respectively. Model predictions are in dark blue (thick solid line), *flgM* reporter data are in light blue (thin solid line and shaded area). The parameters were estimated using a multistart global optimization algorithm (see *Methods and materials* in main text for details). The best fit returns the value $Q = 23.1$ for the objective function, for the parameter vector $(k_0, k_1, n, \theta, K) = (10, 580, 1, 224, 14615)$.

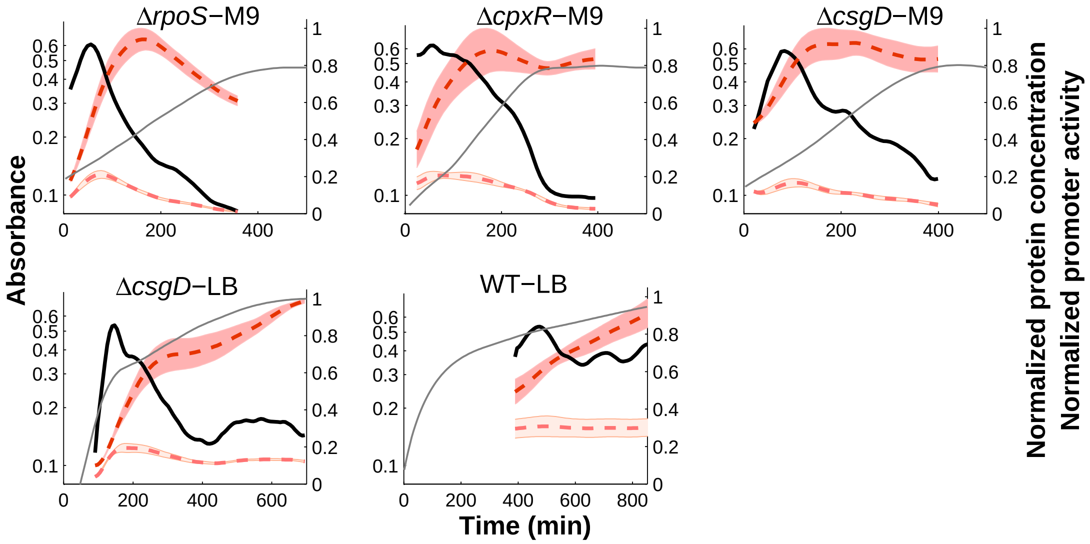

Figure 5. Estimates of FlgM concentrations from reporter gene data. Concentrations of FlgM computed from the *flgM* promoter activity (thick, black solid line) in all experimental conditions considered in this study. The *flgM* activities are the same as shown in Figure 3 of the main text. The dashed, dark red line represents the concentration of the reporter protein, while the dashed, light red line represents the reconstructed FlgM concentration for the measured half-live of 18 min. Promoter activity has been normalized with respect to the maximum of the upper limit of its confidence interval in each condition. All protein concentrations have been normalized with respect to the maximum of the upper limit of the confidence interval of the reporter concentration in each condition. The shaded region corresponds to the mean of the protein concentrations $\pm$ twice the standard error of the mean.

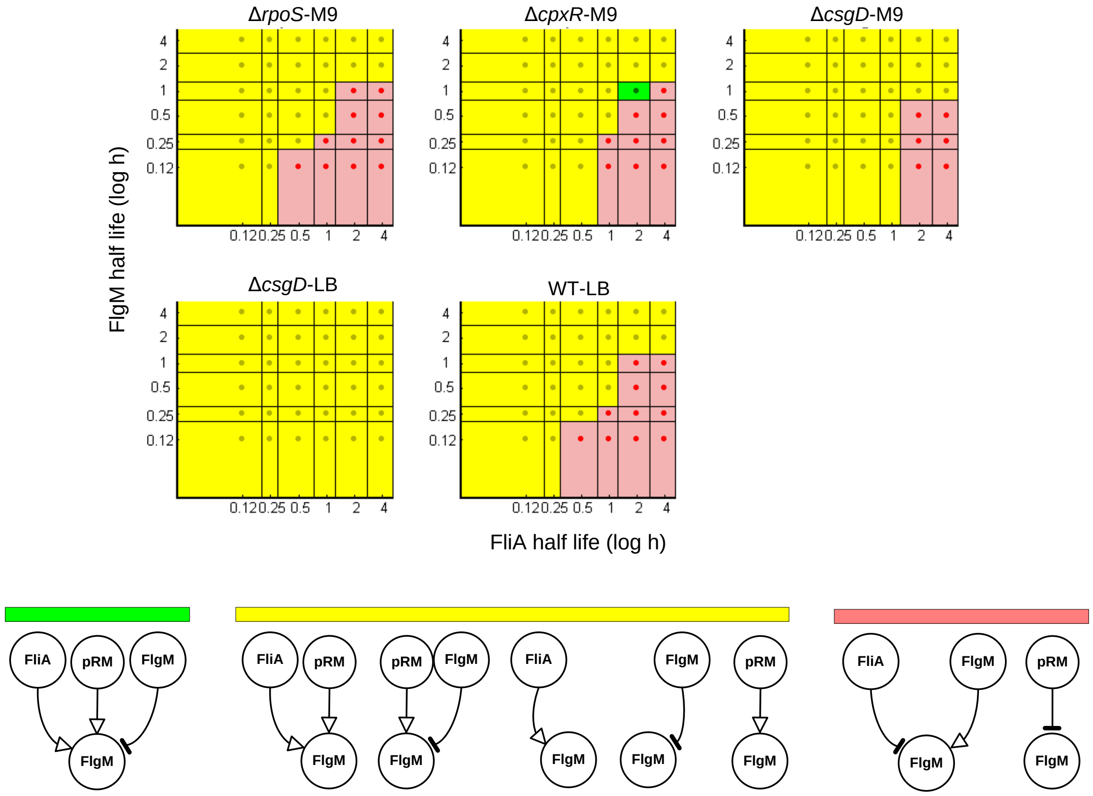

Figure 6. Minimal patterns of regulatory interactions for $flgM$ over a range of physiologically realistic half-lives. The minimal regulatory patterns for the gene *flgM* in the motility network of Figure 5 in the main text as a function of the half-lives of FliA and FlgM. The plots correspond to the five experimental conditions considered (Δ*rpoS*-M9, Δ*cpxR*-M9, Δ*csgD*-M9, Δ*csgD*-LB and WT-LB). The dot in the center of each region in the plots corresponds to a tested combination of half-lives of FliA and FlgM, and thus to specific protein concentration profiles computed from the kinetic model of gene expression (*Methods and materials*). The minimal regulatory patterns were obtained by applying the minimal sign pattern algorithm [1]. The color codes represent the different categories of minimal signal patterns inferred. A region is colored green if the expected regulatory patterns is among the minimal sign patterns returned by the algorithm, and yellow if it is compatible with the returned sign patterns. A region is colored red if none of the returned sign patterns is consistent with the data. Two examples of inconsistent sign patterns are shown.

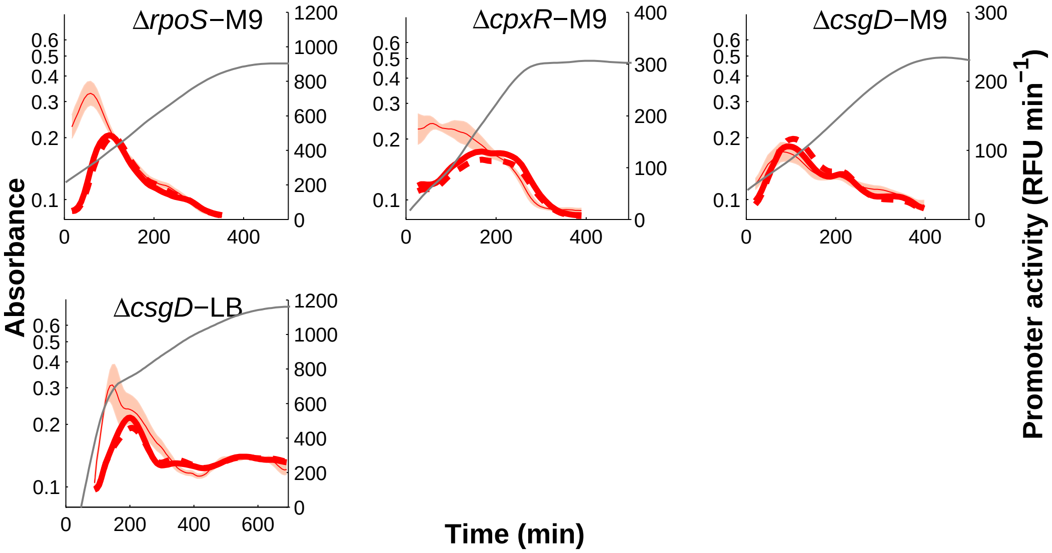

Figure 7. Fits of regulation function of $flgM$ to reporter gene data when reconstructing protein concentrations from the reporter gene data for physiologically realistic half-lives and including global physiological effects. Similarly to Figure 12 of the main text the half-lives have been estimated from the data, within a physiologically plausible range. Two examples of fits are shown, namely the best fit for estimated half-lives of FliA and FlgM (solid line, $Q = 26.2$, $(k_0, k_1, n, \theta, K) = (0.37, 110, 1.4, 24565, 2081)$) and another example of a high-ranking fit. In the case of the best fit obtained, the half-lives of FliA are equal to $(18, 27, 15, 30)$ min in the ($\Delta rpoS$, $\Delta cpxR$, $\Delta csgD$-M9, $\Delta csgD$-LB) conditions, respectively, and the half-lives of FlgM equal to $(7, 7, 7, 30)$ min, respectively.

References

1. Porreca R, Cinquemani E, Lygeros J, Ferrari-Trecate G (2010) Identification of genetic network dynamics with unate structure. Bioinformatics 26: 1239-45.
