# Supplementary material for: Inference of Quantitative Models of Bacterial Promoters from Time-Series Reporter Gene Data
Source: PLoS Comput Biol. 2015 Jan 15;11(1):e1004028. doi: 10.1371/journal.pcbi.1004028 (PMC4295839; doi:10.1371/journal.pcbi.1004028)
Supplement: S8 Text — (PDF) [file pcbi.1004028.s008.pdf]

Supplementary Text S8: Initial conditions for computing protein concentrations[a]

Diana Stefan[1,2], Corinne Pinel[1,2], Stéphane Pinhal[1,2], Eugenio Cinquemani[1], Johannes Geiselmann[1,2], Hidde de Jong[1,*]

1 INRIA Grenoble – Rhône-Alpes, Grenoble, France

2 Laboratoire Interdisciplinaire de Physique (LIPhy, CNRS UMR 5588), Université Joseph Fourier, Grenoble, France

∗ Corresponding author. INRIA Grenoble—Rhône-Alpes, 655 avenue de l'Europe, Monbonnot, 38334 Saint-Ismier Cedex, France. Tel.: +33476615335, Fax: +33456527120, Email: Hidde.de-Jong@inria.fr

As described in the *Methods and materials* section in the main text, the initial condition required for integrating the equation defining the protein concentration of interest is given by Eq. 8. In the text following this equation, some particularities for computing the initial concentration in the case of the FliA-FlgM module are listed. First, the FlgM and FliA half-lives in stationary phase (when no FlgM secretion occurs and FliA is stabilized by FlgM binding) are generally different from the half-lives during exponential growth in the motility-inducing conditions considered here (when FlgM secretion occurs and free FliA is actively degraded by proteases). Second, in rich medium like LB, the activity of *fliA* and *flgM* is negligible during the first few hours of the experiment. The first particularity is addressed by using the protein degradation constant γ'_p, corresponding to the longer half-live in stationary phase, in Eq. 8 in the main text. The second issue is resolved by back-extrapolating the observed promoter activities towards 0. This procedure is illustrated for FliA and FlgM in Figure 1 in this text, in wild-type strain growing in LB

[a]This text contains supplementary information for the paper "Inference of quantitative models of bacterial promoters from time-series reporter gene data".

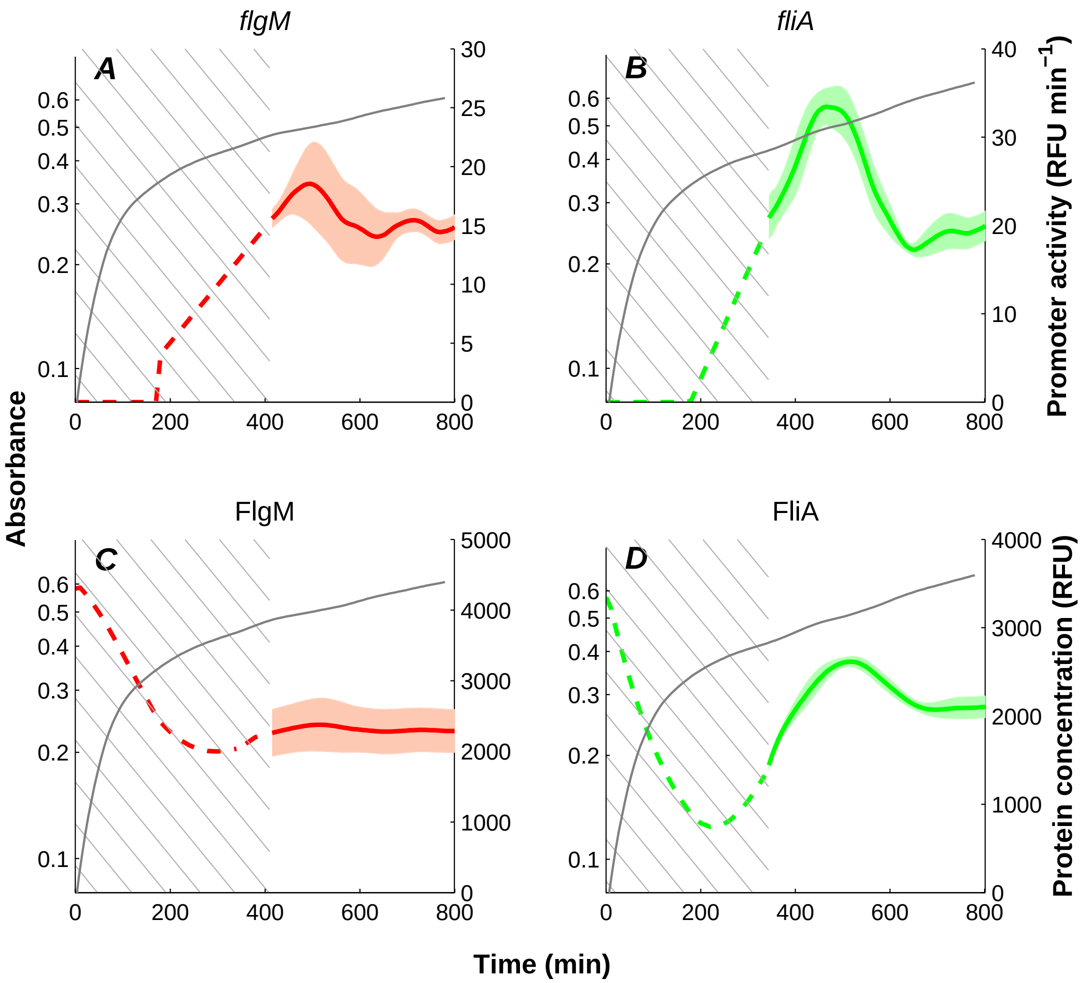

Figure 1. Extrapolation of promoter activities for computing protein concentrations. *A*: The observed promoter activtity of *flgM* (solid line, red) in a wild-type strain growing in LB, and the extrapolation of the observed activity (dashed line, red). *B*: The observed promoter activtity of *fliA* (solid line, green) and its extrapolation (dashed line, green). *C*: Protein concentration of FlgM computed from Eq. 7 in the main text, with a degradation constant γ_p corresponding to 18 min, taking into account the extrapolation of the promoter activity of *flgM* in *A*. The initial concentration was computed from the promoter activity at the end of the preculture, following Eq. 8 in the main text, but using the longer stationary-phase half-live γ_p', corresponding to 3 h for FlgM. *D*: Idem FliA, but with degradation constants γ_p and γ_p' corresponding to half-lives of 30 min and 2 h, respectively. In the hatched regions, the activity of promoters has been extrapolated, *i.e.* $[0, 410]$ min for *flgM* and $[0, 340]$ min for *fliA*. The observed promoter activities have been taken from Figure 3 in the main text. The shaded regions correspond to the mean of the promoter activities and protein concentrations, respectively, $\pm$ twice the standard error of the mean. The absorbance is drawn in solid, grey lines.
