# Supplementary material for: Inference of Quantitative Models of Bacterial Promoters from Time-Series Reporter Gene Data
Source: PLoS Comput Biol. 2015 Jan 15;11(1):e1004028. doi: 10.1371/journal.pcbi.1004028 (PMC4295839; doi:10.1371/journal.pcbi.1004028)
Supplement: S9 Text — (PDF) [file pcbi.1004028.s009.pdf]

Supplementary Text S9: Parameter analysis: computation of active FliA[a]

Diana Stefan[1,2], Corinne Pinel[1,2], Stéphane Pinhal[1,2], Eugenio Cinquemani[1], Johannes Geiselmann[1,2], Hidde de Jong[1,*]
1 INRIA Grenoble – Rhône-Alpes, Grenoble, France
2 Laboratoire Interdisciplinaire de Physique (LIPhy, CNRS UMR 5588), Université Joseph Fourier, Grenoble, France
∗ Corresponding author. INRIA Grenoble—Rhône-Alpes, 655 avenue de l'Europe, Monbonnot, 38334 Saint-Ismier Cedex, France. Tel.: +33476615335, Fax: +33456527120, Email: Hidde.de-Jong@inria.fr

The active regulator in the FliA-FlgM module is free FliA, that is, FliA not bound to FlgM. The active concentration of FliA can be computed from the total concentration of FliA using Eq. 2 in the main text, given a value for the equilibrium constant K and possibly the half-lives of FliA and FlgM, estimated by fitting the model to the *tar* data. This has been done for all situations considered here: (i) replacing protein concentrations by promoter activities; (ii) replacing protein concentrations by promoter activities, while taking into account global physiological effects; (iii) computing protein concentrations for the reference half-lives of FliA and FlgM, while taking into account global physiological effects; (iv) computing protein concentrations for optimized half-lives of FliA and FlgM, while taking into account global physiological effects. The results are shown in Figures 1-4 in this text. Notice that in some of the experimental conditions, FliA is only partially active when protein concentrations instead of promoter activities are used.

[a]This text contains supplementary information for the paper "Inference of quantitative models of bacterial promoters from time-series reporter gene data".

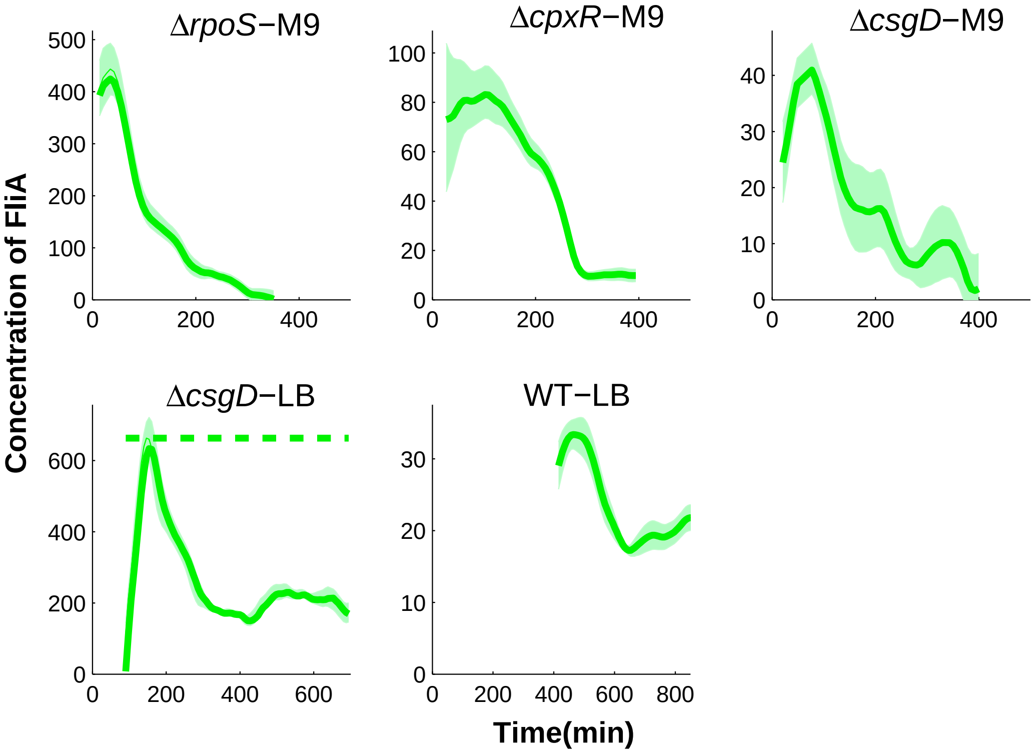

Figure 1. Free and total concentration of FliA when using promoter activities. The concentration of free FliA (solid line, green) is computed by means of Eq. 2 of the main text, for the optimal fit shown in Figure 5 in the main text. The shaded regions represent the confidence intervals of total FliA and correspond to the mean of the promoter activities for 6 replicates $\pm$ twice the standard error of the mean. The threshold parameter θ is shown as a dashed green line.

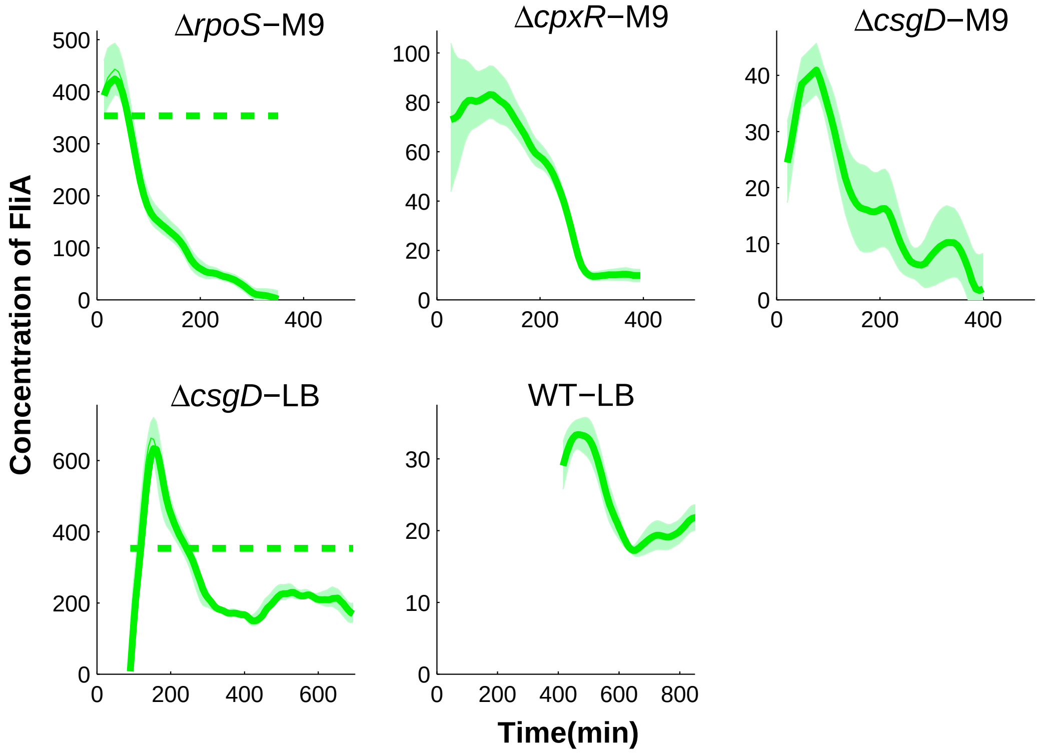

Figure 2. Free and total concentration of FliA when using promoter activities and including global physiological effects. The concentration of free FliA (solid line, green) is computed by means of Eq. 2 of the main text, for the optimal fit shown in Figure 8 in the main text. The shaded regions represent the confidence intervals of total FliA and correspond to the mean of the promoter activities for 6 replicates $\pm$ twice the standard error of the mean. The threshold parameter θ is shown as a dashed green line.

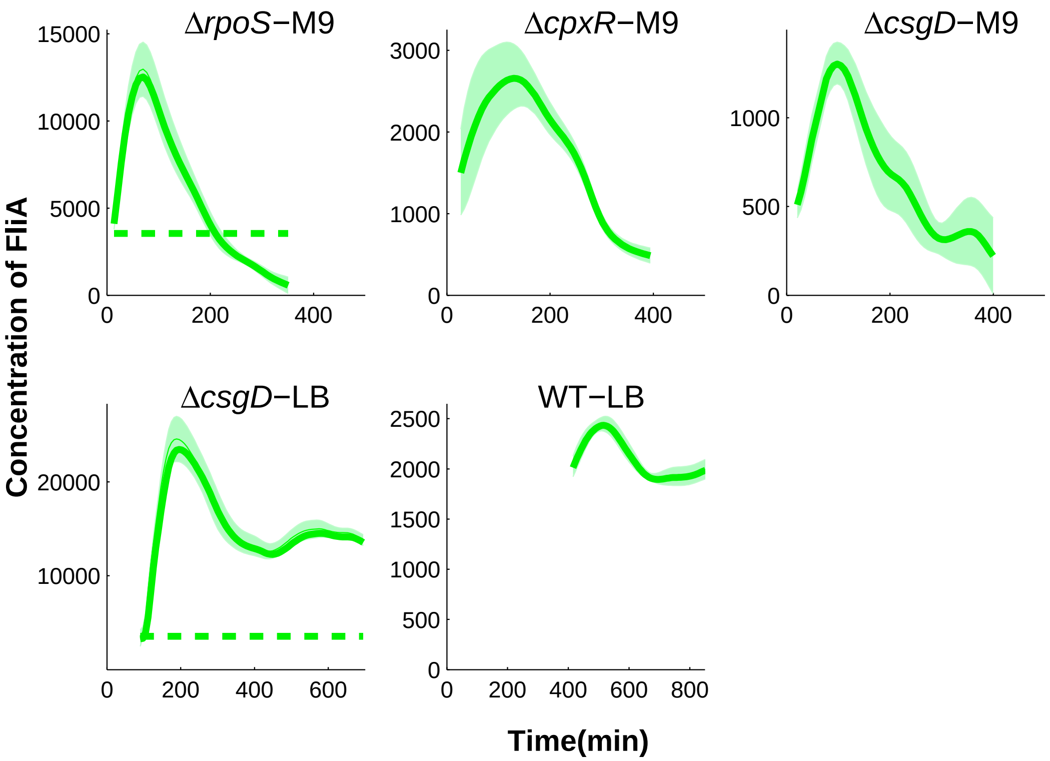

Figure 3. Free and total concentration of FliA when using reconstructed protein concentrations for the measured reference half-lives, and including global physiological effects. The concentration of free FliA (solid line, green) is computed by means of Eq. 2 of the main text, for the optimal fit shown in Figure 10 in the main text. The shaded regions represent the confidence intervals of total FliA and correspond to the mean of the reconstructed protein concentrations for 6 replicates $\pm$ twice the standard error of the mean. The threshold parameter θ is shown as a dashed green line.

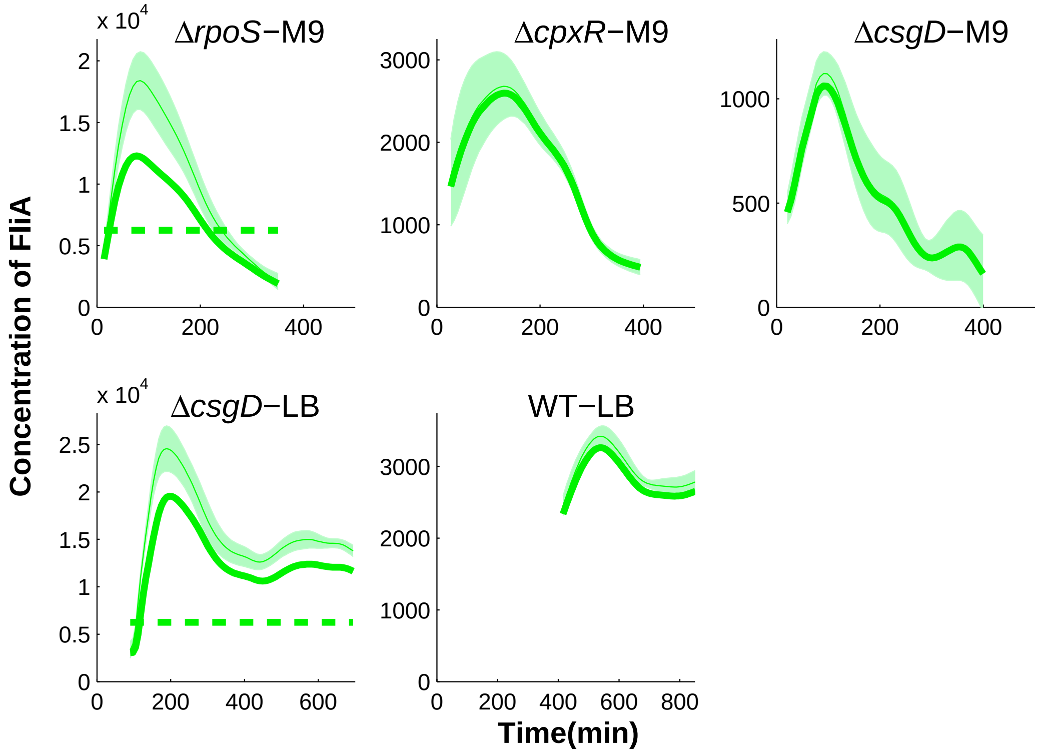

Figure 4. Free and total concentration of FliA when using reconstructed protein concentrations for physiologically-realistic half-lives estimated from the data, and including global physiological effects for tar regulation function. The concentration of free FliA (solid line, green) is computed by means of Eq. 2 of the main text, for the optimal fit shown in Figure 12 in the main text. The shaded regions represent the confidence intervals of total FliA and correspond to the mean of the reconstructed protein concentrations for 6 replicates $\pm$ twice the standard error of the mean. The threshold parameter θ is shown as a dashed green line.
