# Supplementary material for: Inference of Quantitative Models of Bacterial Promoters from Time-Series Reporter Gene Data
Source: PLoS Comput Biol. 2015 Jan 15;11(1):e1004028. doi: 10.1371/journal.pcbi.1004028 (PMC4295839; doi:10.1371/journal.pcbi.1004028)
Supplement: S10 Text — (PDF) [file pcbi.1004028.s010.pdf]

Supplementary Text S10: Computation of parameter confidence intervals[a]

Diana Stefan[1,2], Corinne Pinel[1,2], Stéphane Pinhal[1,2], Eugenio Cinquemani[1], Johannes Geiselmann[1,2], Hidde de Jong[1,*]

1 INRIA Grenoble – Rhône-Alpes, Grenoble, France

2 Laboratoire Interdisciplinaire de Physique (LIPhy, CNRS UMR 5588), Université Joseph Fourier, Grenoble, France

∗ Corresponding author. INRIA Grenoble—Rhône-Alpes, 655 avenue de l'Europe, Monbonnot, 38334 Saint-Ismier Cedex, France. Tel.: +33476615335, Fax: +33456527120, Email: Hidde.de-Jong@inria.fr

As described in the *Methods and materials* section, we use optimization algorithms to estimate numerical values for the parameters occurring in the models of the activity of FliA-dependent promoters (Eqs. 1-2 and Eqs. 2-3 in the main text). In order to obtain confidence intervals for these parameter estimates, we have used a simple procedure analogous to bootstrapping. We use the same notation below as in the *Parameter estimation* subsection of the *Methods and materials*.

The basic idea underlying the procedure is that, at each time point t, we resample the promoter activities derived from the fluorescence data from their own confidence interval. More precisely, given measurements $\big(\bar{x}^s(t), \bar{f}^s(t)\big)$ of $\big(x(t), f(t)\big)$ at times $t \in \mathscr{T}^s$ along with confidence intervals $\big(\bar{x}^s(t) \pm \lambda^s(t), \bar{f}^s(t) \pm \epsilon^s(t)\big)$ (with λ^s equal to twice the standard error of the mean $\bar{x}^s$ and ϵ^s equal to twice the standard error of the mean $\bar{f}^s$), we define new datapoints

$$\big(\bar{x}^s(t) + \kappa^s(t), \bar{f}^s(t) + \delta^s(t)\big), \tag{1}$$

with $\kappa^s(t) \sim N(0, \lambda^s(t)/2)$, $\delta^s(t) \sim N(0, \epsilon^s(t)/2)$, and confidence intervals $\big(\bar{x}^s(t) + \kappa^s(t) \pm \lambda^s(t), \bar{f}^s(t) + \delta^s(t) \pm \epsilon^s(t)\big)$. We thus generate 1000 new datasets. The gene regulation function is then successively fitted against each of these resampled datasets in exactly the same way as described in the main text, yielding an optimal parameter estimate $\hat{c}^i$ for the ith dataset. We define the interval $\hat{C}$ as bounded by 1.5 IQR below the lower quartile and 1.5 IQR above the upper quartile of the set of all bootstrap estimates $\{\hat{c}^i\}_{i=1,\ldots,1000}$, where IQR denotes the interquartile range. In the case of normally distributed data, this interval covers more than 99% of the data. We will use $\hat{C}$ as the confidence interval for the estimate $\hat{c}$ obtained from the original dataset.

Figure 1 in this text shows the confidence intervals for the estimates of the parameters $c = (k_0, k_1, n, \theta, K)$ in the different situations considered in this paper, using promoter activities or protein concentrations for the regulators, and taking into account or ignoring global physiological effects (corresponding to the Figures 5, 8, 10, and 12 in the main text). The results are represented in the form of boxplots summarizing the sets $\{\hat{c}^i\}_{i=1,\ldots,1000}$, normalized with respect to $\mathrm{median}\big(\{\hat{c}^i\}_{i=1,\ldots,1000}\big)$, in each case. As can be immediately seen, for all parameters the confidence interval is small (< 2-fold relative variation). In particular, this implies that the parameters are identifiable, *i.e.*, that they can be unambiguously inferred from the data and, therefore, that the identification problem defined by Eq. 9 in the main text is well-posed.

[a] This text contains supplementary information for the paper "Inference of quantitative models of bacterial promoters from time-series reporter gene data".

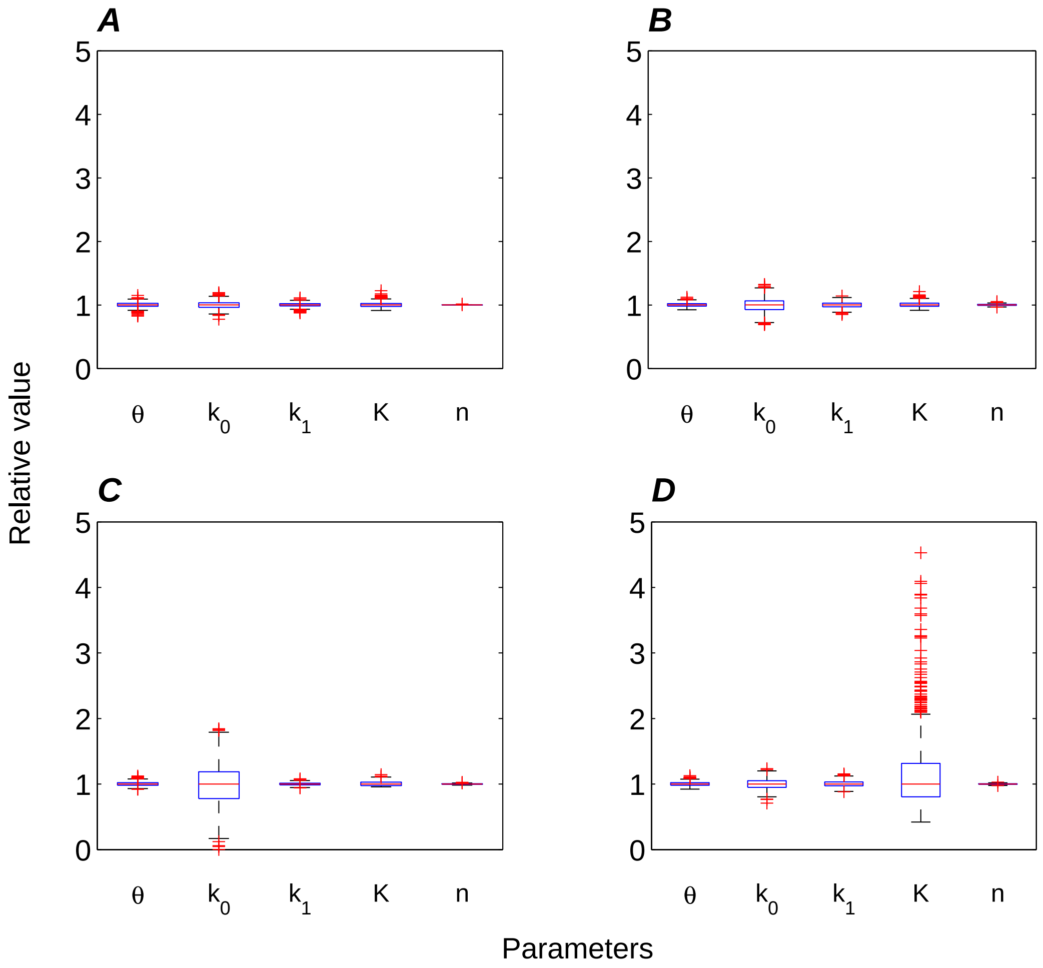

Figure 1. Confidence intervals for the parameters in the promoter activity models. The boxplots represent the results of the bootstrap-like procedure used to estimate confidence intervals, by summarizing for each parameter the estimates from 1000 resampled datasets relative to the median of these estimates. The confidence intervals are indicated by the whiskers of the boxplot, corresponding to 1.5 IQR below the lower quartile and 1.5 IQR above the upper quartile (see the `boxplot` function in MATLAB). *A:* Confidence intervals for the parameter set in Figure 5 in the main text ($Q = 33.4$). *B:* Idem for Figure 8 ($Q = 30.9$). *C:* Idem for Figure 10 ($Q = 25.5$). *D:* Idem for Figure 12 ($Q = 21.0$).
